# Publisher Correction: The β20–β21 of gp120 is a regulatory switch for HIV-1 Env conformational transitions

**DOI:** 10.1038/s41467-017-02463-7

**Published:** 2018-01-08

**Authors:** Alon Herschhorn, Christopher Gu, Francesca Moraca, Xiaochu Ma, Mark Farrell, Amos B. Smith, Marie Pancera, Peter D. Kwong, Arne Schön, Ernesto Freire, Cameron Abrams, Scott C. Blanchard, Walther Mothes, Joseph G. Sodroski

**Affiliations:** 1 Department of Cancer Immunology and Virology, Dana-Farber Cancer Institute, Boston, MA 02215 USA; 2 Department of Microbiology and Immunobiology, Harvard Medical School, Boston, MA 02115 USA; 3 Department of Chemical and Biological Engineering, Drexel University, Philadelphia, PA 19104 USA; 4 Department of Microbial Pathogenesis, Yale University School of Medicine, New Haven, CT 06536 USA; 5 Department of Chemistry, University of Pennsylvania, Philadelphia, PA 19104 USA; 6 Vaccine Research Center, National Institute of Allergy and Infectious Diseases, National Institutes of Health, Bethesda, MD 20892 USA; 7 Department of Biology, Johns Hopkins University, Baltimore, MD 21218 USA; 8 Department of Physiology and Biophysics, Weill Cornell Medical College of Cornell University, New York, NY 10065 USA; 9 Department of Immunology and Infectious Diseases, Harvard T.H. Chan School of Public Health, Boston, MA 02115 USA

Correction to: *Nature Communications* 10.1038/s41467-017-01119-w, published online 19 October 2017

The original version of this Article contained an error in the spelling of the author Amos B. Smith, III, which was incorrectly given as Amos B. SmithIII. This has now been corrected in both the PDF and HTML versions of the Article.

