# Supplementary material for: The β20–β21 of gp120 is a regulatory switch for HIV-1 Env conformational transitions
Source: Nat Commun. 2017 Oct 19;8:1049. doi: 10.1038/s41467-017-01119-w (PMC5648922; doi:10.1038/s41467-017-01119-w)
Supplement: Supplementary file 1 — Supplementary Information [file 41467_2017_1119_MOESM1_ESM.pdf]

Supplementary Methods

Chemical synthesis. *N,N*-diisopropyl ethylamine (0.6 mmol), 4-dimethylaminopyridine (0.03 mmol) and *N*-(3-dimethylaminopropyl)-*N′*-ethylcarbodiimide hydrochloride (0.45 mmol) were added to a solution of carboxylic acid (0.3 mmol) and amine (0.3 mmol) in anhydrous dichloromethane (2 mL). The reaction was stirred at room temperature for 5 hours and subsequently concentrated in vacuo. The resulting residue was purified by flash chromatography to provide the desired compounds. The purity of all compounds was greater than 95 % according to NMR analysis. The chemical scheme for each compound is shown below.

484-3/MF1213

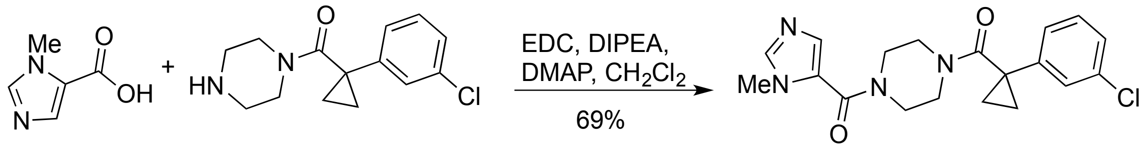

^{1}H NMR (500 MHz, $CDCl_3$) δ = 7.33 – 7.20 (m, 2H), 7.18 (t, *J*=1.8, 0H), 7.09 (d, *J*=7.5, 0H), 3.96 (s, 1H), 3.84 – 3.38 (m, 3H), 1.58 – 1.34 (m, 1H), 1.37 – 1.15 (m, 1H); ^{13}C NMR (126 MHz, $CDCl_3$) δ 170.9, 163.8, 142.3, 135.2, 130.4, 127.3, 125.7, 123.8, 67.5, 29.3, 15.2; IR: 3398, 2983, 2253, 1627, 1242, 733 cm^{-1}; HRMS (ESI) m/z 395.1255 [calcd for $C_{19}H_{21}N_4O_2ClNa$ $(M)^+$ 395.1251].

484-4/MF1216

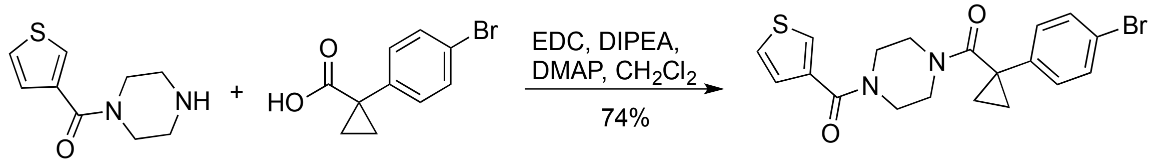

^{1}H NMR (500 MHz, $CDCl_3$) δ = 7.51 (s, 0H), 7.45 (d, *J*=8.3, 1H), 7.39 – 7.31 (m, 0H), 7.14 (d, *J*=4.7, 0H), 7.07 (d, *J*=8.1, 1H), 3.92 – 2.97 (m, 4H), 1.61 – 1.32 (m, 1H), 1.20

(s, 1H); ^{13}C NMR (126 MHz, $CDCl_3$) δ 171.0, 166.2, 139.5, 135.7, 132.2, 127.3, 127.0, 126.5, 120.6, 29.2, 15.2; IR: 3399, 3009, 2924, 2250, 1630, 1431, 1199, 908, 733 cm $^{-1}$; HRMS (ESI) m/z 419.0428 [calcd for $C_{19}H_{20}N_2O_2SBr$ $(M)^+$ 419.0429].

<u>484-11/MF1238</u>

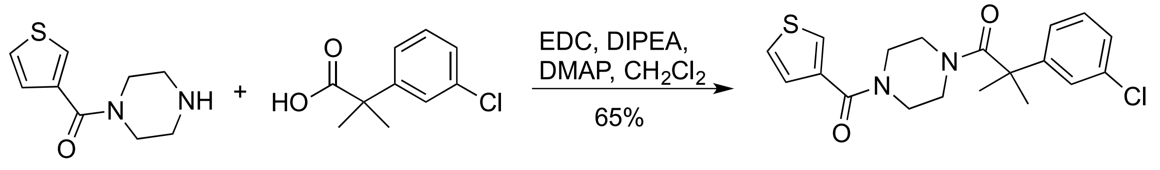

^1H NMR (500 MHz, $CDCl_3$) δ = 7.48 (s, 1H), 7.37 – 7.21 (m, 5H), 7.12 (d, *J*=6.3, 2H), 4.22 – 2.85 (m, 9H), 1.55 (s, 7H); ^{13}C NMR (126 MHz, $CDCl_3$) δ 174.6, 166.0, 148.2, 135.8, 135.2, 130.5, 127.1, 127.1, 127.0, 126.4, 125.0, 123.3, 47.2, 28.3; IR: 3585, 2985, 2253, 1641, 1383, 1096, 907, 733cm^{-1}; HRMS (ESI) m/z 775.1932 [calcd for $C_{38}H_{42}N_4O_4S_2Cl_2$ $(M)^{2+}$ 775.1922].

<u>484-12/MF1239</u>

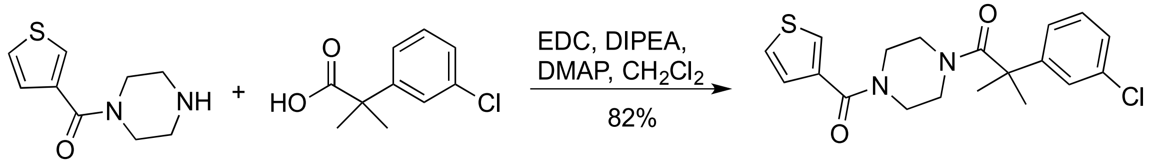

^1H NMR (500 MHz, $CDCl_3$) δ = 7.48 (s, 0H), 7.34 (d, *J*=8.1, 0H), 7.18 (d, *J*=8.4, 0H), 7.12 (d, *J*=4.6, 0H), 3.91 – 2.99 (m, 1H), 1.54 (s, 1H); ^{13}C NMR (126 MHz, $CDCl_3$) δ 174.8, 166.0, 144.6, 135.8, 132.7, 129.4, 127.1, 127.0, 126.4, 126.3, 46.9, 28.4; IR: 3398, 2917, 1631, 1428,m 1257, 1009, 736 cm^{-1}; HRMS (ESI) m/z 377.1074 [calcd for $C_{19}H_{22}N_2O_2SCl$ $(M)^+$ 377.1091].

<u>484-13/MF1240</u>

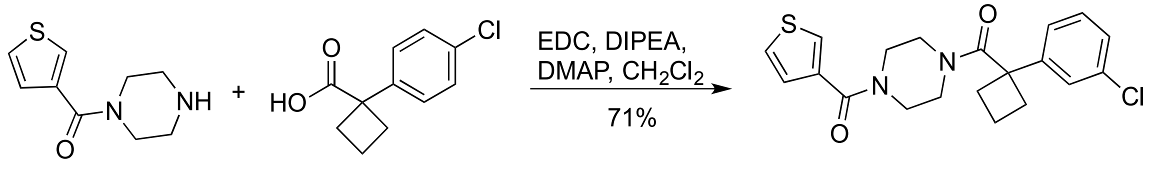

^{1}H NMR (500 MHz, $CDCl_3$) δ = 7.51 (s, 0H), 7.40 – 7.33 (m, 0H), 7.30 – 7.19 (m, 1H), 7.14 (d, *J*=4.9, 1H), 7.08 (d, *J*=7.5, 1H), 3.88 – 2.80 (m, 4H), 2.22 – 1.87 (m, 0H), 1.62 – 1.31 (m, 1H), 1.22 (s, 1H); ^{13}C NMR (126 MHz, $CDCl_3$) δ 170.8, 166.1, 142.6, 135.8, 135.1, 130.3, 127.1, 127.1, 127.0, 126.5, 125.5, 125.5, 123.8, 29.4, 15.4; IR: 3398, 2874, 2254, 1632, 1432, 1012, 733 cm^{-1}; HRMS (ESI) m/z 389.1099 [calcd for $C_{20}H_{22}N_2O_2SCl$ $(M)^+$ 389.1091].

484-15/MF1244

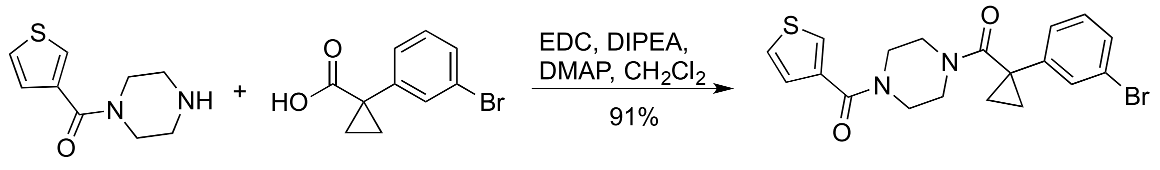

^{1}H NMR (500 MHz, $CDCl_3$) δ = 7.52 (s, 1H), 7.38 – 7.29 (m, 3H), 7.19 (t, *J*=7.8, 1H), 7.16 – 7.09 (m, 2H), 3.92 – 3.02 (m, 8H), 1.51 – 1.34 (m, 2H), 1.22 (s, 2H); ^{13}C NMR (126 MHz, $CDCl_3$) δ 170.9, 166.3, 142.7, 135.5, 130.6, 130.0, 128.4, 128.4, 127.3, 126.92, 126.6, 124.3, 123.3, 29.3, 15.4; IR: 3399, 2983, 2253, 1626, 1425, 1243, 1014, 734cm^{-1}; HRMS (ESI) m/z 419.0434 [calcd for $C_{19}H_{20}N_2O_2SBr$ $(M)^+$ 419.0429].

484-17/MF1246

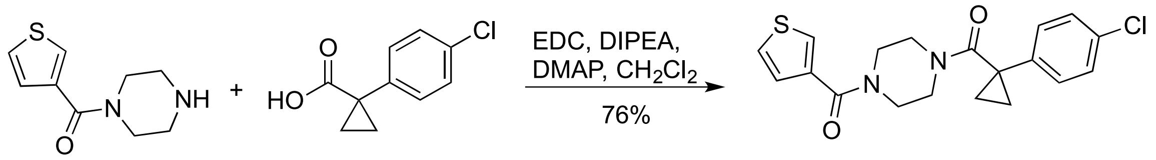

^{1}H NMR (500 MHz, $CDCl_3$) δ = 7.50 (s, 1H), 7.36 – 7.32 (m, 1H), 7.29 – 7.27 (m, 1H), 7.15 – 7.09 (m, 3H), 3.85 – 3.04 (m, 9H), 1.53 – 1.34 (m, 2H), 1.19 (s, 2H); ^{13}C NMR (126 MHz, $CDCl_3$) δ = 171.0, 171.0, 166.2, 139.0, 135.8, 132.6, 129.2, 127.1, 127.0, 126.9, 126.5, 29.2, 15.2; IR: 3444, 2861, 1633, 1428, 1257, 1011, 732 cm^{-1}; HRMS (ESI) m/z 397.0737 [calcd for $C_{19}H_{19}N_2O_2SClNa$ $(M)^+$ 397.0753].

484-18/MF1250

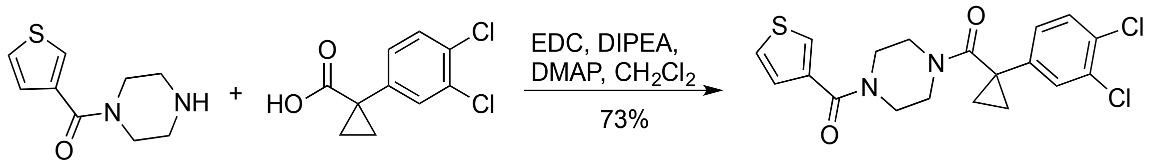

^{1}H NMR (500 MHz, $CDCl_3$) δ = 7.53 (s, 1H), 7.41 – 7.33 (m, 2H), 7.25 (s, 1H), 7.14 (d, *J*=4.8, 1H), 7.07 – 6.99 (m, 1H), 3.85 – 3.33 (m, 8H), 1.50 – 1.42 (m, 2H), 1.21 (s, 2H); ^{13}C NMR (126 MHz, $CDCl_3$) δ = 170.6, 166.5, 140.7, 135.2, 133.3, 131.1, 131.0, 127.5, 127.4, 127.4, 126.9, 126.7, 125.1, 29.0, 15.5; IR: 3399, 1778, 1631, 1200, 1012, 909, 732 cm^{-1}; HRMS (ESI) m/z 409.0539 [calcd for $C_{19}H_{19}N_2O_2SCl_2$ $(M)^+$ 409.0544].

Supplementary Tables

Supplementary Table 1. Inhibition of HIV-1 infection by *N,N'*-difunctionalized piperazines

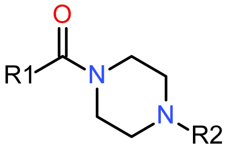

ID	R1	R2	IC_{50} JR-FL (μM)[a]	IC_{50} A-MLV (μM)[a]	Therapeutic Index
110	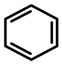	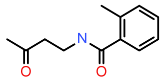	>112	>112	
111	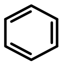	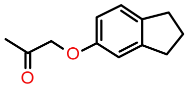	13.2 ± 7.5	>112	>8
112	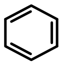	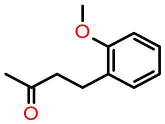	~112	>112	
113	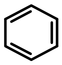	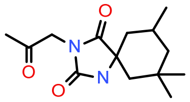	>112	>112	
114	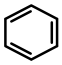	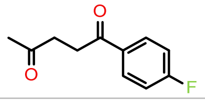	>112	>112	
115	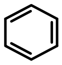	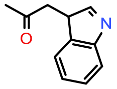	5.5 ± 1.2	>112	>20
116	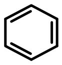	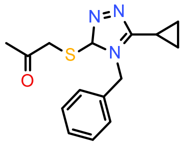	>112	>112	
117	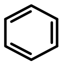	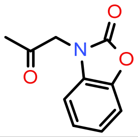	>112	>112	
118	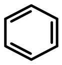	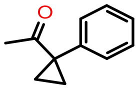	4.7 ± 3.5	>112	>20
119	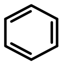	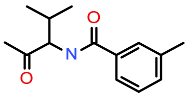	>112	>112	
120	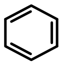	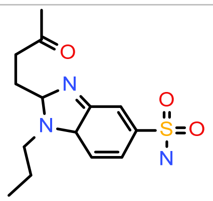	>112	>112	

121	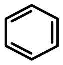	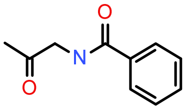	>112	>112	
122	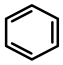	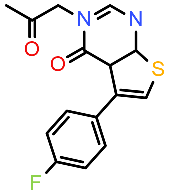	>112	>112	
127	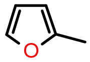	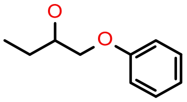	>112	>112	
128	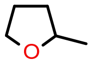	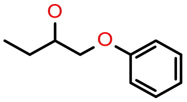	>112	>112	

[a]The ability of the compounds to inhibit the single-round infection of recombinant luciferase-expressing HIV-1 pseudotyped with the HIV-1_{JR-FL} Env or the Env from the control amphotropic murine leukemia virus (A-MLV) was tested. Compounds were initially tested using JC53 target cells[15] and inhibition of active compounds was confirmed using Cf2Th-CD4/CCR5 cells. Inhibition data from two independent experiments, performed in duplicate with Cf2Th-CD4/CCR5 cells, were averaged and the IC_{50} values were calculated by fitting the data to the four-parameter logistic equation.

Supplementary Table 2. Structure-activity relationships of Compound 118 analogues

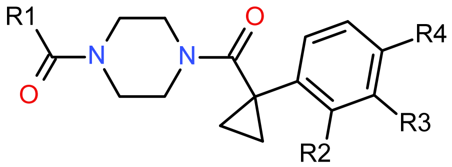

ID	R1	R2	R3	R4	IC_{50} JR-FL (μM)[a]	IC_{50} A-MLV (μM)[a]	Therapeutic Index
242	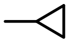	H	H	H	>112	>112	
243	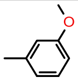	H	H	H	>112	>112	
244	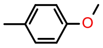	H	H	H	>112	>112	
245	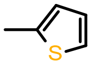	H	H	H	8.6 ± 3.2	>112	>13
246	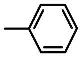	H	H	Br	0.7 ± 0.2	>112	>160
247	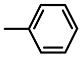	H	Cl	H	1.2 ± 0.2	>112	>93
248	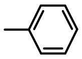	H	CF_3	H	1.4 ± 0.5	>112	>80
249	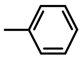	H	H	Cl	4.0 ± 1.7	>112	>28
250	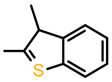	H	H	H	102.4 ± 35.0	>112	>1
252	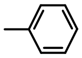	H	Br	H	1.1 ± 0.1	>112	>101
257	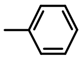	H	OCH_3	CH_3	3.4 ± 0.8	>112	>32
258	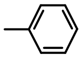	OCH_3	H	H	51.7 ± 4.5	>112	>2
261	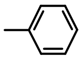	CH_3	H	H	>112	>112	
262	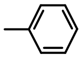	H	H	F	6.0 ± 4.8	>112	>18
263	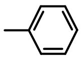	F	H	H	61.2 ± 39.7	>112	>1.8

[a]The ability of the compounds to inhibit the infection of recombinant luciferase-expressing HIV-1 pseudotyped with the HIV-$1_{JR\text{-}FL}$ Env or the A-MLV Env was tested. Compounds were tested once with JC53 target cells and at least once with Cf2Th-CD4/CCR5 cells. Similar inhibition efficiency was observed with both cells. Inhibition data from two independent experiments (for compounds **245**, **249** and **252**) or a

single experiment (all the rest), all performed in duplicate with Cf2Th-CD4/CCR5 cells, were averaged. IC_{50} values were calculated by fitting the data to the four-parameter logistic equation.

Supplementary Table 3. Structure-activity relationships of Compound 484 analogues

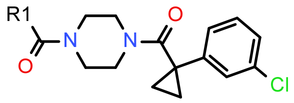

ID	R1	R2	R3	R4	IC_{50} JR-FL (μM)[a]	IC_{50} A-MLV (μM)[a]	Therapeutic Index
480	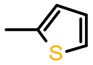	H	H	Br	1.0 ± 0.2	>112	>112
481	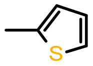	H	Cl	H	1.5 ± 0.2	>112	>75
482	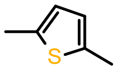	H	Cl	H	15.3 ± 2.0	>112	>7
483	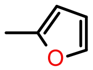	H	Cl	H	3.2 ± 0.7	>112	>35
484	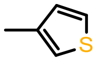	H	Cl	H	0.4 ± 0.1	>112	>280
485	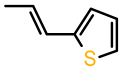	H	Cl	H	73.9 ± 12.5	67.3 ± 10.1	0.9
486	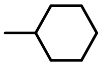	H	Cl	H	21.9 ± 6.8	>112	>5
487	-$CH(CH_2CH_3)_2$	H	Cl	H	68.6 ± 14.4	>112	>1.6
488	-$CH_2CH_2CH_3$	H	Cl	H	>112	>112	
489	-$CH_2CH(CH_3)_2$	H	Cl	H	>112	>112	
490	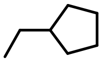	H	Cl	H	62.4 ± 13.1	>112	>1.7
491	-$CH(CH_3)_3$	H	Cl	H	35.4 ± 10.2	>112	>3
492	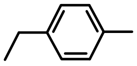	H	Cl	H	96.2 ± 8.6	>112	>1.2
493	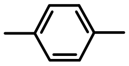	H	Cl	H	24.8 ± 11.5	>112	>4
494	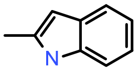	H	Cl	H	2.0 ± 0.2	2.7 ± 1.2	1.4
495	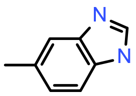	H	Cl	H	>112	>112	

[a]The ability of the compounds to inhibit the infection of recombinant luciferase-expressing HIV-1 with the HIV-1$_{JR\text{-}FL}$ or A-MLV Env was tested. Inhibition data from 2-3 independent experiments (for compounds **480-484**) or a single experiment (all the rest), all performed in duplicate with Cf2Th-CD4/CCR5 cells, were averaged. IC_{50} values were calculated by fitting the data to the four-parameter logistic equation.

Supplementary Table 4. The effect of 484 and DMJ-II-121 on infectivity of different HIV-1 mutants

	Region	Secondary structure	484		DMJ-II-121	
			IC_{50} [μM][a]	Fold change	IC_{50} [μM][a]	Fold change
JR-FL						
WT			0.40 ± 0.10	1	15.04	1
D107R	C1	α1	5.40 ± 1.08	13.5	2.4 ± 0.4	0.2
I109W	C1	α1	1.39 ± 0.27	3.5	32.6 ± 2.3	2.2
W112A	C1	α1	11.56 ± 5.75	28.9	>100	> 6.7
Q114E	C1	α1	0.81 ± 0.06	2.0	42.6 ± 2.6	2.8
V134A	V1		0.63 ± 0.03	1.6	6.3 ± 0.2	0.4
N139A	V1		0.33 ± 0.04	0.8	16.8 ± 1.2	1.1
M147A	V1		0.24 ± 0.15	0.6	7.7 ± 0.6	0.5
E153A	V1		0.65 ± 0.11	1.6	3.1 ± 0.1	0.21
I154A	V1		14.85 ± 2.91	37.1	0.29 ± 0.15	0.02
K155A	V1		2.54 ± 0.31	6.4	ND	
N156A			6.00 ± 2.72	15	0.19 ± 0.08	0.01
R166A	V2		0.68 ± 0.04	1.7	ND	
Y173A	V2		0.41 ± 0.10	1.0	3.1 ± 0.2	0.21
L175A	V2		0.58 ± 0.05	1.5	0.85 ± 0.11	0.06
Y177A	V2		13.40 ± 1.64	33.5	0.69 ± 0.12	0.05
K178A	V2		0.36 ± 0.02	0.9	ND	
L193A[b]	V2		> 112	> 280	0.06 ± 0.004	0.004
I194A	V2		0.25 ± 0.02	0.6	ND	
T320E	V3		0.62 ± 0.03	1.6	ND	
S375W[b]	C3		> 112	> 280	> 100	> 6.7
Q422A	C4	β20	3.08 ± 0.86	7.7	2.42 ± 0.09	0.2
I423A[b]	C4	β20	41.48 ± 5.10	103	2.29 ± 0.51	0.2
I424A	C4	β20	14.54 ± 2.62	36.4	30.24 ± 7.34	2
M426L[b]	C4		32.95 ± 3.03	82.4	21.8 ± 2.7	1.5
M426A	C4		0.01 ± 0.001	0.03	31.77 ± 8.14	2.1
Q428A	C4		0.33 ± 0.01	0.8	> 100	> 6.7
V430A	C4	β21	ND		> 100	> 6.7
K432A	C4	β21	0.60 ± 0.02	1.5	18.21 ± 2.64	1.2
M434A[b]	C4	β21	1.36 ± 0.50	3.4	5.12 ± 2.0	0.3
Y435A	C4	β21	4.97 ± 0.82	12.4	> 100	> 6.7
Y435K	C4	β21	94.66 ± 10.82	236.6	59.1 ±6.8	4
W479A	C5	α5	0.22 ± 0.01	0.6	> 100	> 6.7
ADA						
WT			27.82 ± 2.33		1.6 ± 0.14	
ΔV1V2	V1/V2		>112	>4	0.063 ± 0.047	0.04

Sensitive WT Resistant

[a] Recombinant HIV-1 pseudotyped with the indicated wild-type (WT) or mutant HIV-$1_{JR\text{-}FL}$ Envs was tested for inhibition by **484** or the CD4-mimetic compound DMJ-II-121.The IC_{50} values and fold change (relative to WT Env) associated with moderate and high resistance are highlighted in orange and red,

respectively; those associated with moderate and large increases in sensitivity are highlighted in blue and green, respectively. IC_{50} values were calculated by fitting the average inhibition data from 2-3 independent experiments, most of them performed in triplicate, to a four-parameter logistic equation.

b Highly resistant mutants to BMS-806 (fold change: I423A > 1000, S375W >1000, L193A=75. M426L and M434A were previously reported to confer resistance to BMS-806[16-18])

c Interestingly, the S375W change, which fills the Phe 43 cavity with the indole ring of the substituted tryptophan residue,[20] resulted in complete resistance to both inhibitors as well as to BMS-806. The Phe 43 cavity-filling S375W substitution may sterically impede 484 binding or decrease HIV-1 sensitivity to conformational blockers by increasing Env sampling of the CD4-bound conformation.[19,20] The deletion of the V1/V2 region, another alteration that increases Env sampling of the CD4-bound conformation,[21] also resulted in decreased HIV-1 sensitivity to 484.

Supplementary Table 5. Docking scores and IC_{50} values of 484-related compounds

Compounds	Structure	IC_{50} JR-FL (μM)	Docking Score[a]	
			Glide	MM-GBSA (kcal mol^{-1})
484-18	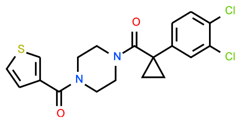	0.08	-11.48	-40.20
484-17	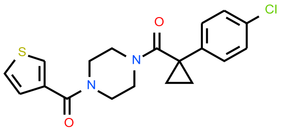	0.16	-11.31	-38.84
484	Supplementary Table 3	0.4	-11.16	-43.15
484-13	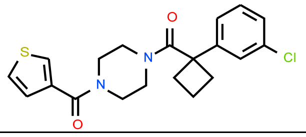	0.4	-11.22	-39.58
484-4	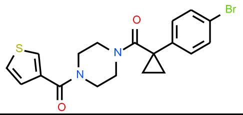	0.4	-11.23	-38.84
484-15	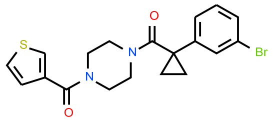	0.5	-11.03	-39.43
252	Supplementary Table 2	1.0	-11.22	-43.74
480		1.0	-11.08	-45.49
481	Supplementary Table 3	1.5	-10.88	-38.40
483		3.2	-10.72	-38.20
249	Supplementary Table 2	4.0	-11.33	-45.49
118	Supplementary Table 1	4.7	-11.08	-40.64
115		5.5	-11.22	-36.73
484-11	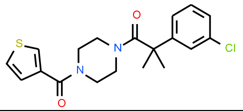	>112	-10.99	-36.54
484-12	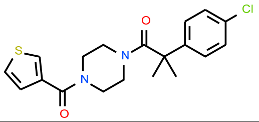	>112	-10.67	-36.37
484-3	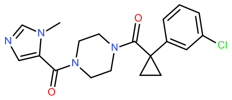	>112	-9.05	-33.59

[a] The IC_{50} values of the compounds for inhibition of HIV-$1_{JR\text{-}FL}$ infection of Cf2Th-CD4/CCR5 cells are shown. Docking score calculation is described in the Methods section; inactive compounds are shown in red.

Supplementary Table 6. Antibodies used in this study

Ligand	Group	Target
19b	Antibodies that recognize CD4-bound Env conformations	gp120 V3 loop[1]
17b		A discontinuous epitope that contains residues of the β2, β3, β20, and β21 sheets[2]
902090		A linear epitope composed of residues 171-177[3]
830A		V2i (V2-integrin) antibody, contacts residues R153, V154, T175, Y177, L179, D180 and I194 on the surface of a gp120 V2 β-barrel[4]
F105	Weakly neutralizing	Weakly neutralizing CD4-binding site antibody[5,6]
VRC01<br>VRC03<br>3BNC117	broadly neutralizing antibodies (bNAbs)	CD4-binding site[7,8]
PG9		Quaternary V2 gp120 epitope[9]
VRC34		gp120-gp41 hybrid epitope[10]
10-1074<br>PGT121		V3-directed antibody; binding depends on the presence of glycosylation at Asn 332[7,11]
10E8<br>7H6<br>4E10		Membrane-proximal external region of gp41 (MPER)[12-14]

Supplementary Table 7. Primary HIV-1 strains that contain amino acids other than leucine at position 193 and the related β20-β21 amino acid sequence

	Residue 191-201 (V2)	Residue 420-435 (β20-β21)
**JR-FL (reference)**	YR**L**ISCDTSVI	IKQIINMWQEVGKAMY
A1D.UG.2011.DEURF11UG006.KF716485	YR**I**INCNTSAI	IKQIINMWQ**RA**GKAMY
01_AE.CN.2007.LN070008.JX112854	YR**I**INCNTSVI	I**R**QIINMWQ**GA**G**Q**AMY
01_AE.TH.-.NP03.AB485654	YR**I**INCNSSVI	IKQIINMWQ**GA**G**Q**AMY
01_AE.CN.2010.CYM059.JX112796	YR**I**TSCNTSVI	IKQ**FV**NMWQ**G**VG**Q**AMY
01_AE.CN.2010.10LNA103.JX960608	YR**I**INCNTSVI	IKQI**VR**MWQ**G**VG**Q**AMY
B.CO.2001.PCM074.AY561240	YR**M**ISCNTSSV	IKQIINMWQEVGKAMY
BC.IN.2002.NARI9-3.EU000508	YR**M**INCNTSVI	IKQIINMWQEVG**R**AMY
BC.IN.2002.INDNARI_0218440.EU000514	YR**M**IHCNTSTI	IKQI**V**NMWQEVG**R**AMY
BC.MM.1999.mIDU103.AB097873	YR**M**INRNTSVI	IKQ**VV**NMWQEVGKAMY
B.TH.2004.04TH601066.JN248329	YR**M**IHCNTSVI	IRQI**V**NMWQEVGKAMY
B.US.-.CR0382N.FJ469725	YT**V**ISCNTSVI	IKQ**F**IN**R**WQEVGKAMY
B.US.2007.07US_SAJ_C166_MS.JF689886	YI**I**RSCNTSVI	IKQIINMWQEVGKAMY
B.JP.2011.DEMB11JP002.KF716497	YR**I**INCNTSVI	IKQIINMWQ**T**VGKAMY
B.US.2006.CR0276Z.FJ469714	YR**V**ISCNTSVI	IKQ**F**IN**R**WQEVGKAMY
B.ES.2006.X2102.EU786677	YR**V**ISCNTSVI	IKQ**F**INMWQEVGKAMY
B.US.2006.06US_SAJ_C165_TJ.JF689863	YR**I**INCNTSVI	IKQIIN**L**WQEVGKAMY
B.AR.1998.ARCH054.AY037268	YR**I**KSCNTSVI	IKQ**F**INMWQ**K**VGKAMY
B.US.2007.HIV_US_BID-V3044_2007.JQ403066	YR**V**ISCNTSVI	IKQIINMWQEVGKAMY
B.KR.2003.03HJY8.JQ316131	YT**M**INCNSSAI	IK**H**IIN**R**WQEVGKAMY
B.CY.2008.CY237.JF683784	YR**M**TSCNTSVI	IRQI**V**N**L**WQEVGKAMY
B.US.2005.USPI38417EI33y05051pcWG2B2.JN024363	YR**M**ISCNTSVI	IRQI**V**N**R**WQEVGKA**I**Y
B.BR.2005.05BR1078.JN692461	YM**M**INCNTSVI	IKQIIN**K**WQEVGKAMY
B.ZA.2003.03ZAPS045MB2.DQ396398	YM**I**RSCNTSVI	IKQ**F**INMWQEVGKAMY
B.BR.2003.BREPM1024.EF637056	YM**I**INCNTSVI	IKQIIN**K**WQEVGKAMY
B.DK.2004.PMVL_018.EF514697	YR**M**ISCNTSVI	IKQIINMWQEVGKAMY
B.PL.-.DEMBXXPL001.KC596069	EP**I**PIHYCAPA	IKQIINMWQ**K**VGKAMY
B.JP.1999.DR1348.AB287370	YR**M**ISCNTSII	IKQIINMWQ**G**VGKAMY
B.US.2010.DEMB10US004.KC473827	YI**M**TSCNTSVL	IKQIINMWQEVGKAMY
B.JP.-.DR1712.AB604946	YR**M**ISCNTSVI	IKQ**V**INMWQEVGKAMY
B.US.-.F7174.DQ886032	YR**M**INCNTTVI	IKQIINMWQEVGRAMY
B.PE.2006.502_0648_FL02.JF320215	YR**I**TSCNTSTI	IKQIINMWQEVGKAMY
B.US.2011.CP12-10.KF384799	YR**I**RSCNTSVI	IKQIINMWQEVGKAMY
BF1.BR.1999.BREPM107.AY771588	YM**I**INCNTSVI	IKQIIN**R**WQEVGKAMY
B.US.2007.HIV_US_BID-V3020_2007.JQ403059	YR**M**INCNTSVI	IKQIINMWQEVGKAMY
C.IN.2005.C.IN.05.NIRT723.1.KF766541	YR**F**INCNTSTI	IKQ**F**INMWQEVG**R**AMY
C.BW.1996.96BW1104.AF110969	YR**F**INCSTSTS	IKQ**F**IN**L**WQEVG**R**AMY
C.BW.1996.96BWM032.AF443075	YR**W**INCNTSSI	IKQIIN**T**WQEVG**R**A**I**Y
C.ZA.2000.1214MB.AY463236	YR**I**ISCNTSTI	IKQ**M**INMWQ**G**VG**R**AMY
D.ZA.1986.R482.AY773341	YR**F**ICCNTSAI	I**R**QII**Y**MWQ**K**VGKAMY
G.CM.2001.A1786.FJ389367	YR**M**INCNVSTI	IKQI**VR**MWQ**R**VG**Q**AMY
O.GA.2011.11Gab6352.JX245015	YT**I**INCNSTTI	**LR**Q**VVRS**W**MGGQSGL**Y
02D.GH.2003.GHNJ193.AB231897	YR**V**INCNTLSH	IKQI**VR**MWQ**R**VG**Q**AMY
02_AG.CM.2001.01CM_0074NY.AY371131	YR**F**INCNTSAI	IKQI**V**NMWQ**K**VG**Q**AMY
11_cpx.FR.1999.MP1307.AJ291720	YN**I**NKCNIVTI	IKQI**VRL**WQ**R**VG**Q**AMY
17_BF.PY.2002.PY02_PSP0096.EU581823	YR**I**IRCNTSTI	IRQ**VV**NMWQEVG**R**AMY
18_cpx.CU.1999.CU68.AY894993	CI**G**INCNVSXI	IKQI**VR**MWQ**RT**G**Q**AMY
19_cpx.CU.1999.CU7.AY894994	-K**I**INCNTSAI	IKQ**VV**NMWQ-VG**Q**AMY
35_AD.IR.2011.11IR.KSH29F.AB703611	YR**V**INCNTSAI	IKQIINMWQ**RT**G**Q**AMY
39_BF.BR.2003.03BRRJ327.EU735536	---TNCNSSTI	IKQI**V**NMWQEVG**R**AMY

Supplementary Table 8. Primers used in the study for site directed mutagenesis

Change	Sequence
**HIV-1$_{JR-FL}$**	
I420A	5'-catcaccctgccttgcagggccaagcagatcatcaacatg-3'
	5'-catgttgatgatctgcttggccctgcaaggcagggtgatg-3'
K421A	5'-caccctgccttgcaggatcgcccagatcatcaacatgtggc-3'
	5'-gccacatgttgatgatctgggcgatcctgcaaggcagggtg-3'
Q422A	5'-cctgccttgcaggatcaaggccatcatcaacatgtggcagg-3'
	5'-cctgccacatgttgatgatggccttgatcctgcaaggcagg-3'
Q422D	5'-ctgccttgcaggatcaaggacatcatcaacatgtggcag-3'
	5'-ctgccacatgttgatgatgtccttgatcctgcaaggcag-3'
Q422E	5'-tgccttgcaggatcaaggagatcatcaacatgtgg-3'
	5'-ccacatgttgatgatctccttgatcctgcaaggca-3'
Q422K	5'-tgccttgcaggatcaagaagatcatcaacatgtgg-3'
	5'-ccacatgttgatgatcttcttgatcctgcaaggca-3'
Q422N	5'-ctgccttgcaggatcaagaacatcatcaacatgtggcag-3'
	5'-ctgccacatgttgatgatgttcttgatcctgcaaggcag-3'
Q422R	5'-ccctgccttgcaggatcaagagaatcatcaacatgtggcagga-3'
	5'-tcctgccacatgttgatgattctcttgatcctgcaaggcaggg-3'
Q422T	5'-cctgccttgcaggatcaagaccatcatcaacatgtggcagg-3'
	5'-cctgccacatgttgatgatggtcttgatcctgcaaggcagg-3'
I423A	5'-ccttgcaggatcaagcaggccatcaacatgtggcagga-3'
	5'-tcctgccacatgttgatggcctgcttgatcctgcaagg-3'
I423F	5'-cttgcaggatcaagcagttcatcaacatgtggcag-3'
	5'-ctgccacatgttgatgaactgcttgatcctgcaag-3'
I423K	5'-cttgcaggatcaagcagaagatcaacatgtggcaggag-3'
	5'-ctcctgccacatgttgatcttctgcttgatcctgcaag-3'
I423L	5'-ccttgcaggatcaagcagctgatcaacatgtggcaggag-3'
	5'-ctcctgccacatgttgatcagctgcttgatcctgcaagg-3'
I423M	5'-tgccttgcaggatcaagcagatgatcaacatgtgg-3'
	5'-ccacatgttgatcatctgcttgatcctgcaaggca-3'
I423V	5'-ccttgcaggatcaagcaggtgatcaacatgtggcaggag-3'
	5'-ctcctgccacatgttgatcacctgcttgatcctgcaagg-3'
I423R	5'-gccttgcaggatcaagcagcggatcaacatgtggcaggagg-3'
	5'-cctcctgccacatgttgatccgctgcttgatcctgcaaggc-3'
I424A	5'-gcaggatcaagcagatcgccaacatgtggcaggagg-3'
	5'-cctcctgccacatgttggcgatctgcttgatcctgc-3'
N425A	5'-ggatcaagcagatcatcgccatgtggcaggaggtgg-3'
	5'-ccacctcctgccacatggcgatgatctgcttgatcc-3'
M426A	5'-gatcaagcagatcatcaacgcctggcaggaggtgggcaagg-3'
	5'-ccttgcccacctcctgccaggcgttgatgatctgcttgatc-3'
W427A	5'-agcagatcatcaacatggcccaggaggtgggcaaggc-3'
	5'-gccttgcccacctcctgggccatgttgatgatctgct-3'
Q428A	5'-cagatcatcaacatgtgggccgaggtgggcaaggccatg-3'
	5'-catggccttgcccacctcggcccacatgttgatgatctg-3'
E429A	5'-tcaacatgtggcaggccgtgggcaaggccatg-3'
	5'-catggccttgcccacggcctgccacatgttga-3'
V430A	5'-aacatgtggcaggaggccggcaaggccatgtatg-3'
	5'-catacatggccttgccggcctcctgccacatgtt-3'
G431A	5'-gtggcaggaggtggccaaggccatgtatg-3'
	5'-catacatggccttggccacctcctgccac-3'

Change	Sequence
**HIV-1$_{\text{JR-FL}}$**	
K432A	5'-gtggcaggaggtgggcgccgccatgtatgctcctc-3'
	5'-gaggagcatacatggcggcgcccacctcctgccac-3'
A433G	5'-ggaggtgggcaagggcatgtatgctcctc-3'
	5'-gaggagcatacatgcccttgcccacctcc-3'
M434A	5'-ggaggtgggcaaggccgcctatgctcctcccatca-3'
	5'-tgatgggaggagcataggcggccttgcccacctcc-3'
Y435A	5'-gtgggcaaggccatggctgctcctcccatcag-3'
	5'-ctgatgggaggagcagccatggccttgcccac-3'
Y435D	5'-gtgggcaaggccatggacgctcctcccatcagg-3'
	5'-cctgatgggaggagcgtccatggccttgcccac-3'
Y435E	5'-gtgggcaaggccatggaggctcctcccatcagg-3'
	5'-cctgatgggaggagcctccatggccttgcccac-3'
Y435F	5'-ggtgggcaaggccatgtttgctcctcc-3'
	5'-ggaggagcaaacatggccttgcccacc-3'
Y435G	5'-ggtgggcaaggccatgggcgctcctcccatcaggg-3'
	5'-ccctgatgggaggagcgcccatggccttgcccacc-3'
Y435H	5'-gtgggcaaggccatgcacgctcctcccatcagg-3'
	5'-cctgatgggaggagcgtgcatggccttgcccac-3'
Y435I	5'-ggtgggcaaggccatgatcgctcctcccatcaggg-3'
	5'-ccctgatgggaggagcgatcatggccttgcccacc-3'
Y435K	5'-gtgggcaaggccatgaaggctcctcccatcagg-3'
	5'-cctgatgggaggagccttcatggccttgcccac-3'
Y435L	5'-ggtgggcaaggccatgctggctcctcccatcaggg-3'
	5'-ccctgatgggaggagccagcatggccttgcccacc-3'
Y435M	5'-ggtgggcaaggccatgatggctcctcccatcaggg-3'
	5'-ccctgatgggaggagccatcatggccttgcccacc-3'
Y435N	5'-gtgggcaaggccatgaacgctcctcccatcagg-3'
	5'-cctgatgggaggagcgttcatggccttgcccac-3'
Y435P	5'-ggtgggcaaggccatgcccgctcctcccatcaggg-3'
	5'-ccctgatgggaggagcgggcatggccttgcccacc-3'
Y435Q	5'-gtgggcaaggccatgcaggctcctcccatcagg-3'
	5'-cctgatgggaggagcctgcatggccttgcccac-3'
Y435R	5'-ggtgggcaaggccatgcgggctcctcccatcaggg-3'
	5'-ccctgatgggaggagcccgcatggccttgcccacc-3'
Y435S	5'-ggtgggcaaggccatgagcgctcctcccatcaggg-3'
	5'-ccctgatgggaggagcgctcatggccttgcccacc-3'
Y435T	5'-ggtgggcaaggccatgaccgctcctcccatcaggg-3'
	5'-ccctgatgggaggagcggtcatggccttgcccacc-3'
Y435V	5'-ggtgggcaaggccatggtggctcctcccatcaggg-3'
	5'-ccctgatgggaggagccaccatggccttgcccacc-3'
Y435W	5'-gaggtgggcaaggccatgtgggctcctccca-3'
	5'-tgggaggagcccacatggccttgcccacctc-3'
**HIV-1$_{\text{BG505}}$**	
I423A	5'-ataactctcccatgcagaataaagcaagccataaatatgtggcagagaataggacaa-3'
	5'-ttgtcctattctctgccacatatttatggcttgctttattctgcatgggagagttat-3'
**HIV-1$_{\text{ZM53M.PB12}}$**	
I423A	5'-acagacatcatactcctatgtagaataaaacaagccataaacatgtggcaggaggtag-3'
	5'-ctacctcctgccacatgtttatggcttgttttattctacataggagtatgatgtctgt-3'
**HIV-1$_{\text{190049}}$**	
I423A	5'-acactcccatgcagaataaaacaagccataaacatgtggcaggg-3'
	5'-ccctgccacatgtttatggcttgttttattctgcatgggagtgt-3'

Supplementary Figures

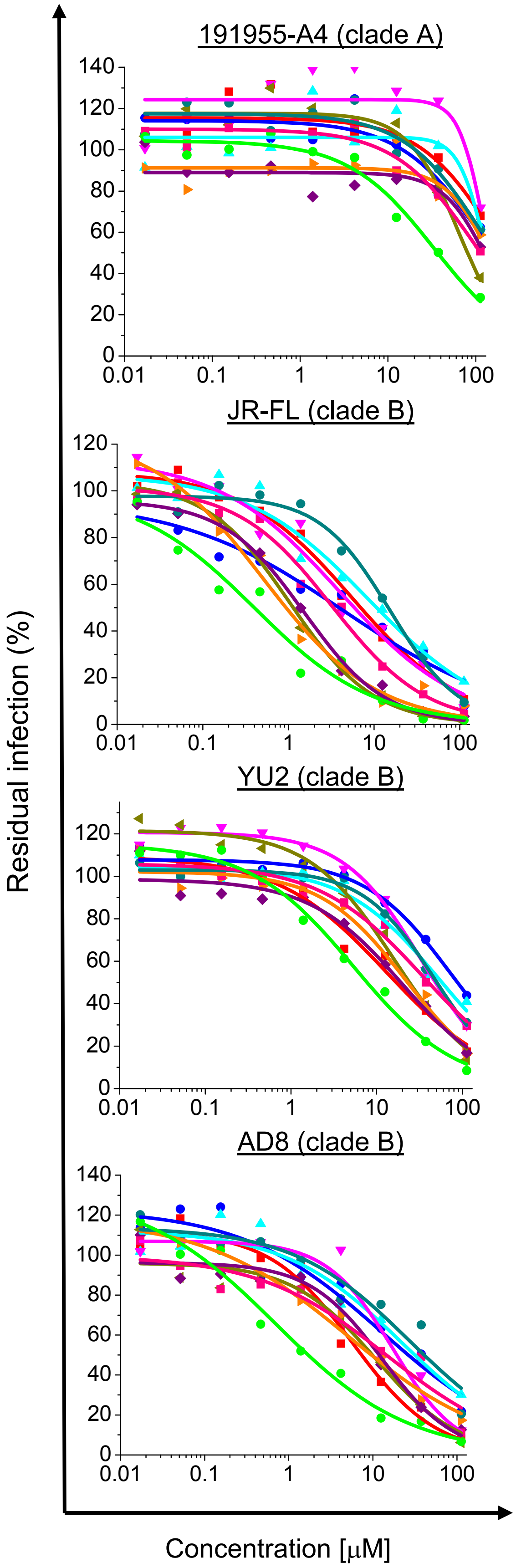

Compound	IC_{50} [μM]	R^2
115	>112	
118	>112	
245	>112	
249	>112	
252	65.7 ± 10.1	0.93
480	>112	
481	>112	
482	>112	
483	93.8 ± 18.7	0.92
484	33.4 ± 5.7	0.96
115	5.5 ± 1.2	0.98
118	4.7 ± 3.5	0.93
245	8.6 ± 3.2	0.95
249	4.0 ± 1.7	0.96
252	1.1 ± 0.1	0.99
480	0.5 ± 0.2	0.98
481	1.5 ± 0.2	0.99
482	15.3 ± 2.0	0.98
483	3.2 ± 0.7	0.98
484	0.4 ± 0.1	0.95
115	13.1 ± 3.3	0.98
118	74.2 ± 8.2	0.95
245	52.0 ± 10.2	0.96
249	33.6 ± 2.9	0.99
252	16.0 ± 2.3	0.98
480	21.8 ± 4.5	0.96
481	19.9 ± 5.1	0.95
482	47.6 ± 4.1	0.99
483	34.5 ± 6.3	0.97
484	5.4 ± 0.8	0.99
115	5.8 ± 1.4	0.97
118	12.8 ± 6.9	0.91
245	22.1 ± 8.6	0.91
249	17.1 ± 4.2	0.93
252	10.8 ± 3.5	0.93
480	5.2 ± 2.1	0.97
481	12.9 ± 3.1	0.95
482	27.6 ± 10.4	0.91
483	14.6 ± 7.4	0.90
484	0.7 ± 0.4	0.97

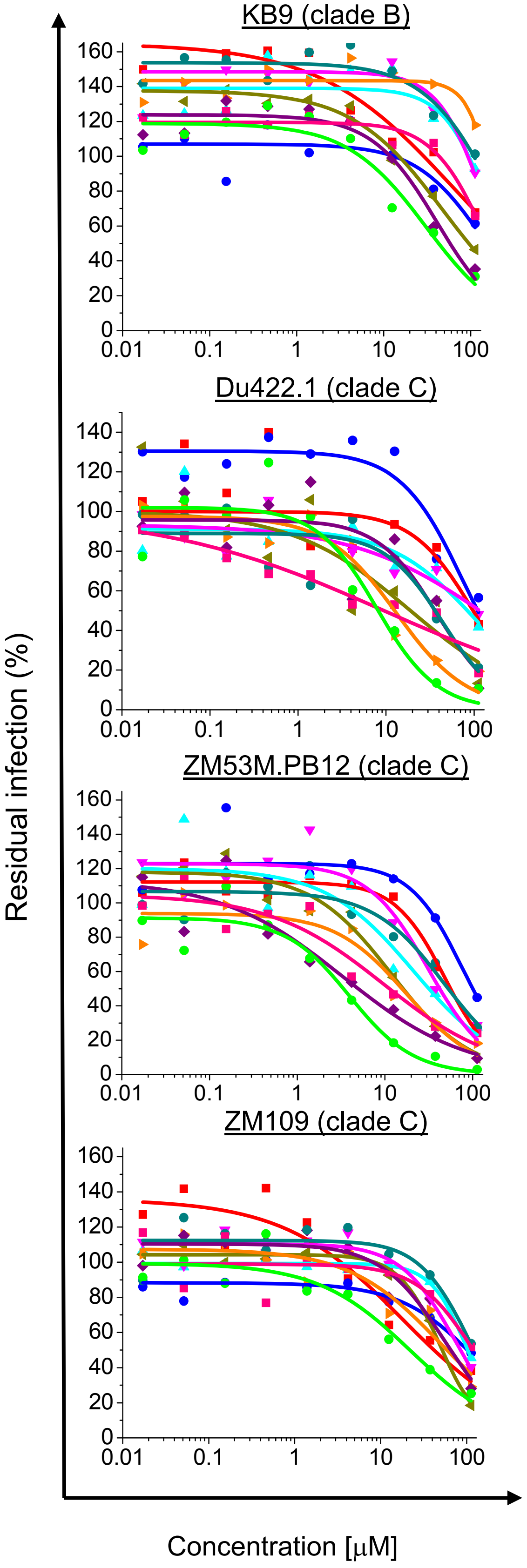

Compound	IC_{50} [μM]	R^2
115	62.0 ± 20.1	0.91
118	>112	
245	>112	
249	>112	
252	48.3 ± 6.5	0.97
480	>112	
481	41.3 ± 7.9	0.93
482	>112	
483	>112	
484	30.7 ± 8.9	0.89
115	97.5 ± 61.5	0.43
118	74.6 ± 18.1	0.89
245	103.3 ± 66.4	0.50
249	>112	
252	18.9 ± 12.8	0.70
480	13.1 ± 4.2	0.90
481	38.6 ± 19.7	0.66
482	43.5 ± 18.2	0.70
483	9.4 ± 3.4	0.90
484	7.9 ± 3.2	0.86
115	48.5 ± 7.1	0.94
118	77.0 ± 23.3	0.73
245	21.4 ± 11.1	0.77
249	31.2 ± 8.5	0.88
252	11.7 ± 2.8	0.96
480	17.7 ± 6.4	0.86
481	3.4 ± 2.6	0.86
482	46.8 ± 15.0	0.83
483	9.8 ± 4.9	0.89
484	4.0 ± 1.4	0.91
115	17.6 ± 8.6	0.87
118	>112	
245	103.9 ± 7.6	0.96
249	73.7 ± 12.2	0.91
252	49.7 ± 3.6	0.98
480	49.8 ± 18.3	0.84
481	55.7 ± 14.0	0.87
482	105.7 ± 29.1	0.71
483	>112	
484	22.2 ± 8.5	0.87

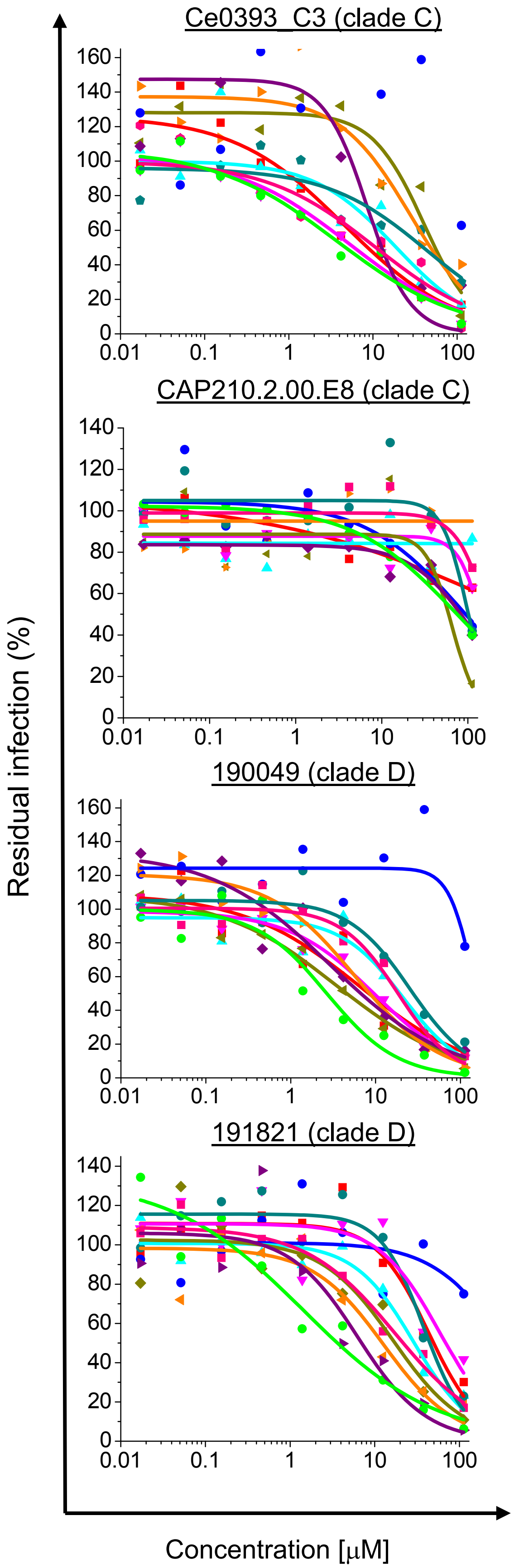

Compound	IC_{50} [μM]	R^2
115	4.6 ± 2.9	0.87
118	>112	
245	18.9 ± 9.6	0.77
249	5.3 ± 2.3	0.95
252	40.0 ± 12.8	0.82
480	27.9 ± 11.9	0.78
481	8.6 ± 4.5	0.67
482	44.4 ± 24.9	0.71
483	10.8 ± 5.1	0.85
484	3.8 ± 2.0	0.94
115	>112	
118	84.3 ± 47.2	0.65
245	>112	
249	>112	
252	65.3 ± 22.2	0.64
480	>112	
481	>112	
482	98.3 ± 22.0	0.62
483	>112	
484	70.5 ± 8.0	0.98
115	6.4 ± 4.6	0.84
118	>112	
245	21.1 ± 7.4	0.97
249	9.8 ± 2.0	0.96
252	3.6 ± 1.4	0.95
480	5.7 ± 1.8	0.90
481	2.9 ± 1.9	0.89
482	25.5 ± 7.1	0.93
483	18.2 ± 4.4	0.88
484	2.7 ± 1.2	0.84
115	43.1 ± 11.3	0.85
118	>112	
245	29.0 ± 6.9	0.91
249	58.6 ± 24.7	0.67
252	16.5 ± 7.9	0.81
480	12.7 ± 5.4	0.84
481	6.3 ± 3.4	0.80
482	38.0 ± 8.5	0.88
483	17.7 ± 5.1	0.93
484	1.4 ± 1.2	0.92

191859 (clade D)

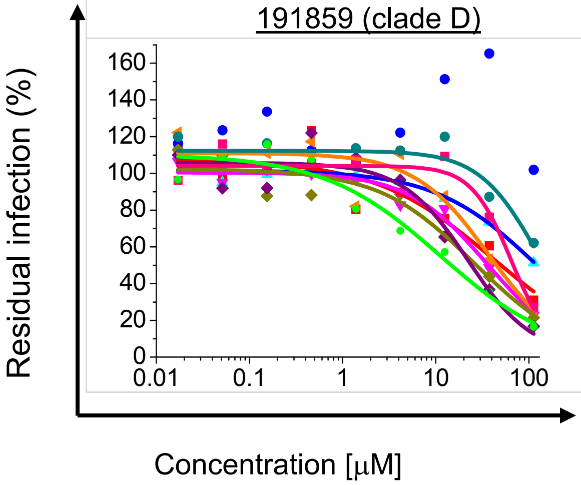

Compound	IC_{50} [μM]	R^2
■ 115	40.8 ± 19.5	0.80
● 118	>112	
▲ 245	>112	
▼ 249	36.3 ± 5.5	0.97
◀ 252	25.9 ± 7.9	0.91
▶ 480	37.3 ± 12.0	0.85
♦ 481	22.1 ± 6.2	0.90
● 482	>112	
■ 483	65.5 ± 11.3	0.89
● 484	11.2 ± 3.9	0.94

Supplementary Figure 1. Inhibition of different HIV-1 strains by selected compounds. Ten related compounds were selected based on their potency against HIV-$1_{JR\text{-}FL}$ for profiling the antiviral activity against recombinant HIV-1 pseudotyped with different HIV-1 Envs. The Envs were derived from diverse laboratory-adapted, transmitted/founder and primary HIV-1 strains. Inhibition data from 2-3 independent experiments, each performed in duplicate, were averaged. IC_{50} values were calculated by fitting the averaged data to the four-parameter logistic equation.

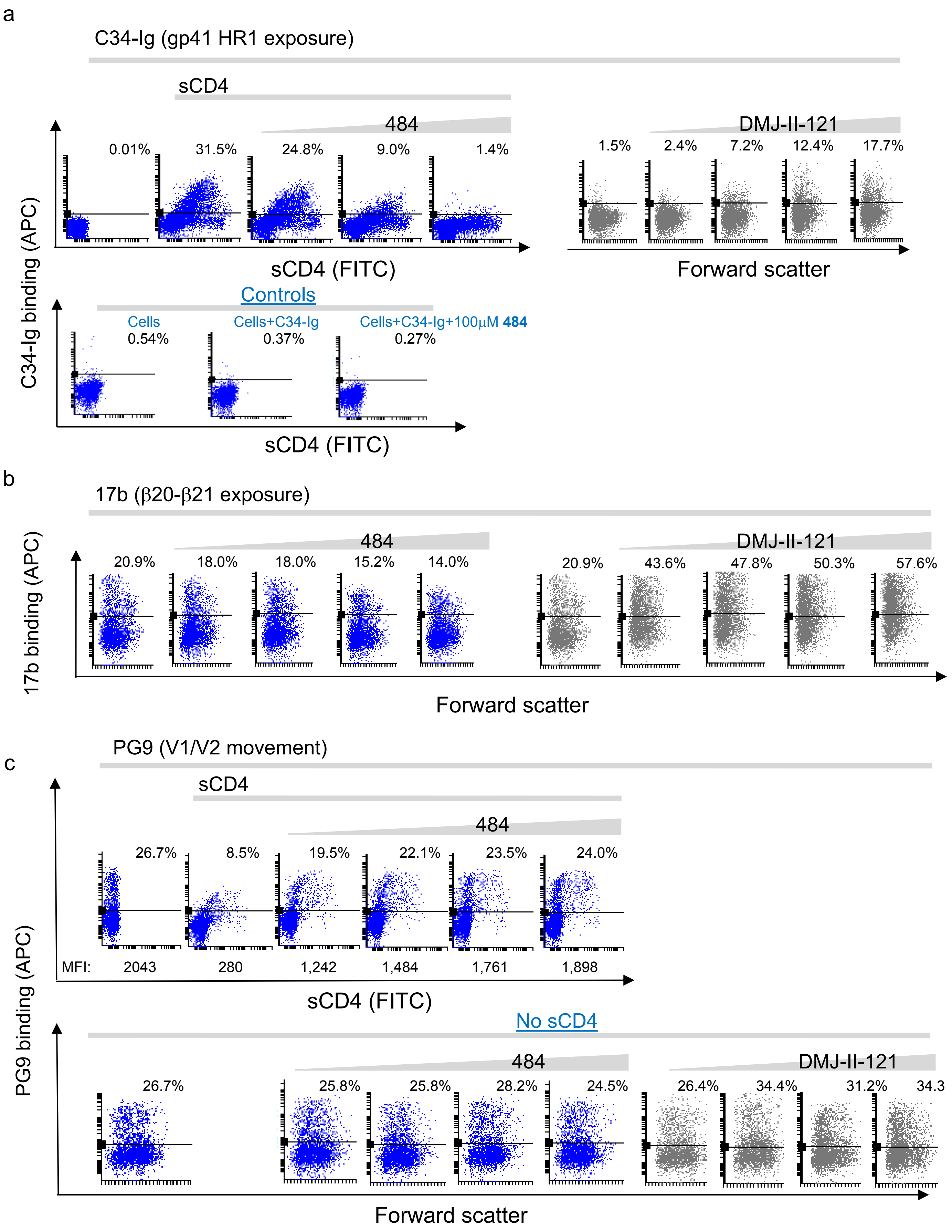
a
C34-Ig (gp41 HR1 exposure)
sCD4
484
DMJ-II-121
0.01%
31.5%
24.8%
9.0%
1.4%
1.5%
2.4%
7.2%
12.4%
17.7%
C34-Ig binding (APC)
sCD4 (FITC)
Forward scatter
Controls
Cells
0.54%
Cells+C34-Ig
0.37%
Cells+C34-Ig+100µM 484
0.27%
sCD4 (FITC)
b
17b (β20-β21 exposure)
484
DMJ-II-121
20.9%
18.0%
18.0%
15.2%
14.0%
20.9%
43.6%
47.8%
50.3%
57.6%
17b binding (APC)
Forward scatter
c
PG9 (V1/V2 movement)
sCD4
484
26.7%
8.5%
19.5%
22.1%
23.5%
24.0%
MFI:
2043
280
1,242
1,484
1,761
1,898
sCD4 (FITC)
PG9 binding (APC)
No sCD4
484
DMJ-II-121
26.7%
25.8%
25.8%
28.2%
24.5%
26.4%
34.4%
31.2%
34.3
Forward scatter

d

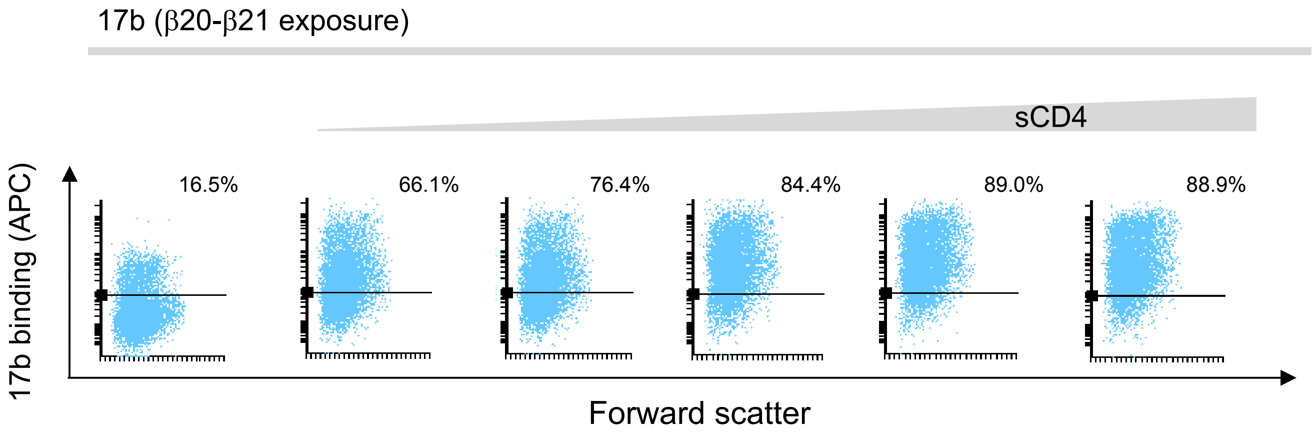

Supplementary Figure 2. Opposing effects of 484 and the CD4-mimetic compound DMJ-II-121 on CD4-induced rearrangements of HIV-1 Env. The effect of the specified small molecules on the binding of C34-Ig (**a**) or the 17b (**b**) or PG9 (**c**) antibodies to the cell-surface HIV-$1_{JR\text{-}FL\Delta CT}$ Env trimer was measured by two-color flow cytometry. Soluble CD4 (sCD4) was added in some cases, as indicated. For PG9 binding, HIV-$1_{JR\text{-}FL\Delta CT}$ E168K+N188A, which restores the PG9 epitope to the HIV-$1_{JR\text{-}FL}$ Env, was used. Compounds were tested at 1, 5, 50 and 100 μM (1, 5 and 50 μM for testing the effects of **484** on C34-Ig binding (**a**, left panel), or as specified) and no cytotoxic effects were observed at these concentrations. (**d**) A control for the experiment shown in panel (b): binding of 17b to HIV-$1_{JR\text{-}FL\Delta CT}$ in the presence of increasing concentrations of sCD4 (1.2, 3.7, 11, 33 and 100 μg/ml), All incubations were done at room temperature to allow efficient Env binding without significant shedding. Results shown are representative of those obtained in 2-3 independent experiments. APC, allophycocyanin; FITC, fluorescein isothiocyanate; MFI, mean fluorescence intensity.

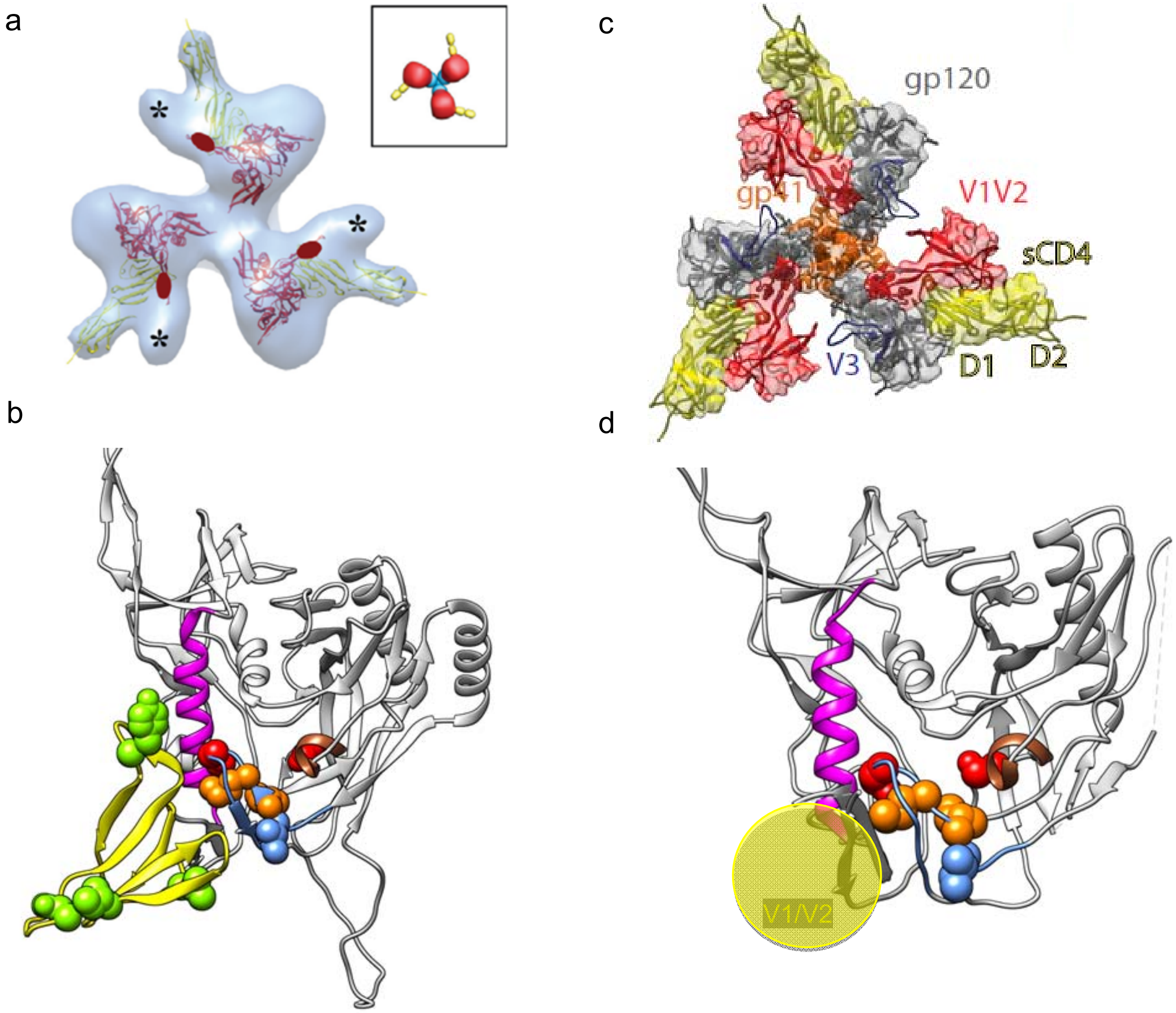

Supplementary Figure 3. Available structures of the CD4-bound conformation of HIV-1 Env. (a) Cryoelectron tomographic map of HIV-1 Env bound to sCD4 and fit with sCD4 and gp120 crystal structures.[22] The sCD4 is shown in yellow and HIV-1 gp120 core in red. Asterisk indicates a density presumably corresponding to the gp120 V1/V2 region and magenta ovals mark the V1/V2 stem. (b) Model of the CD4-bound conformation of gp120 built by a computational protocol that used cryo-electron tomography maps (from a), atomic-resolution structures of the CD4-bound gp120 core, and information on binding interactions (PDB 3J70).[23] **(c)** Density map of HIV-1 Env bound to sCD4 determined by single-particle cryoelectron microscopy (cryo-EM) fit with coordinates of the molecular dynamics model of full-length CD4-bound gp120.[24] **(d)** Structure based on the cryo-EM density map of an HIV-1_{BG505} sgp140 SOSIP.664 Env-sCD4-17b-8ANC195 complex (PDB 5THR; the V1/V2 region is schematically shown). The atoms shown involve amino acid residues whose alteration resulted in changes in 484 or DMJ-II-121 sensitivity, and are colored according to the key in Figure 2c.

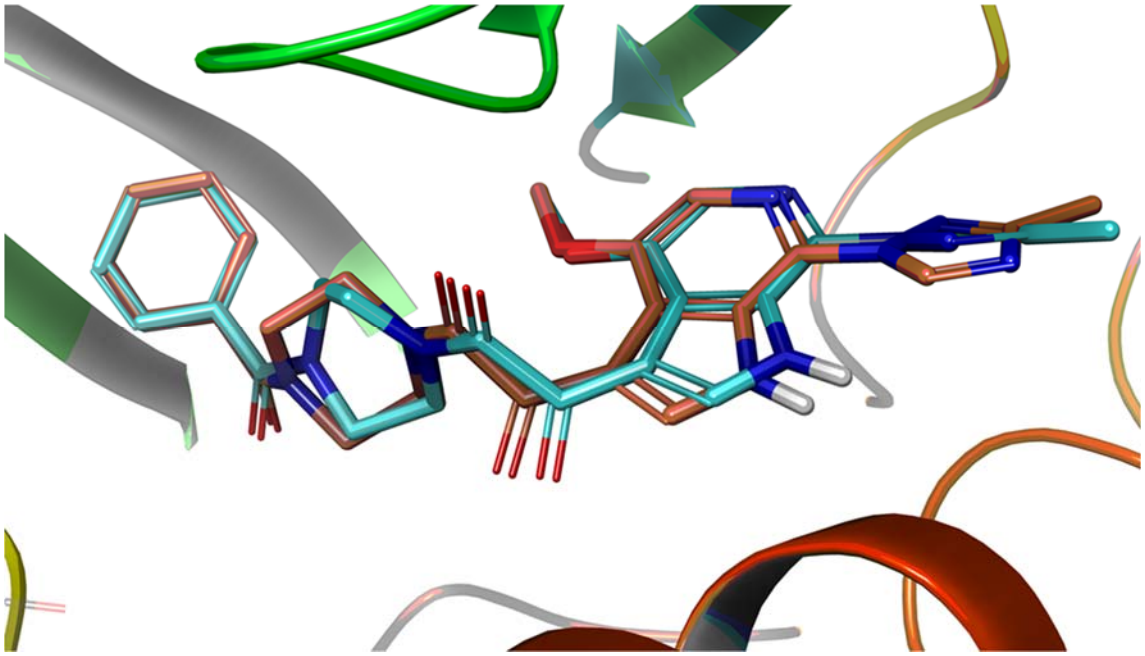

Supplementary Figure 4. Docking the control BMS-626529 compound into the crystal structure of HIV-1$_{BG505}$ sgp140 SOSIP.664. Docking was performed as described in the Methods section and the calculated root mean square distance (RMSD) between the position of BMS-626529 in the crystal structure (cyan sticks) and the position after docking (orange sticks) is 1.0 Å.

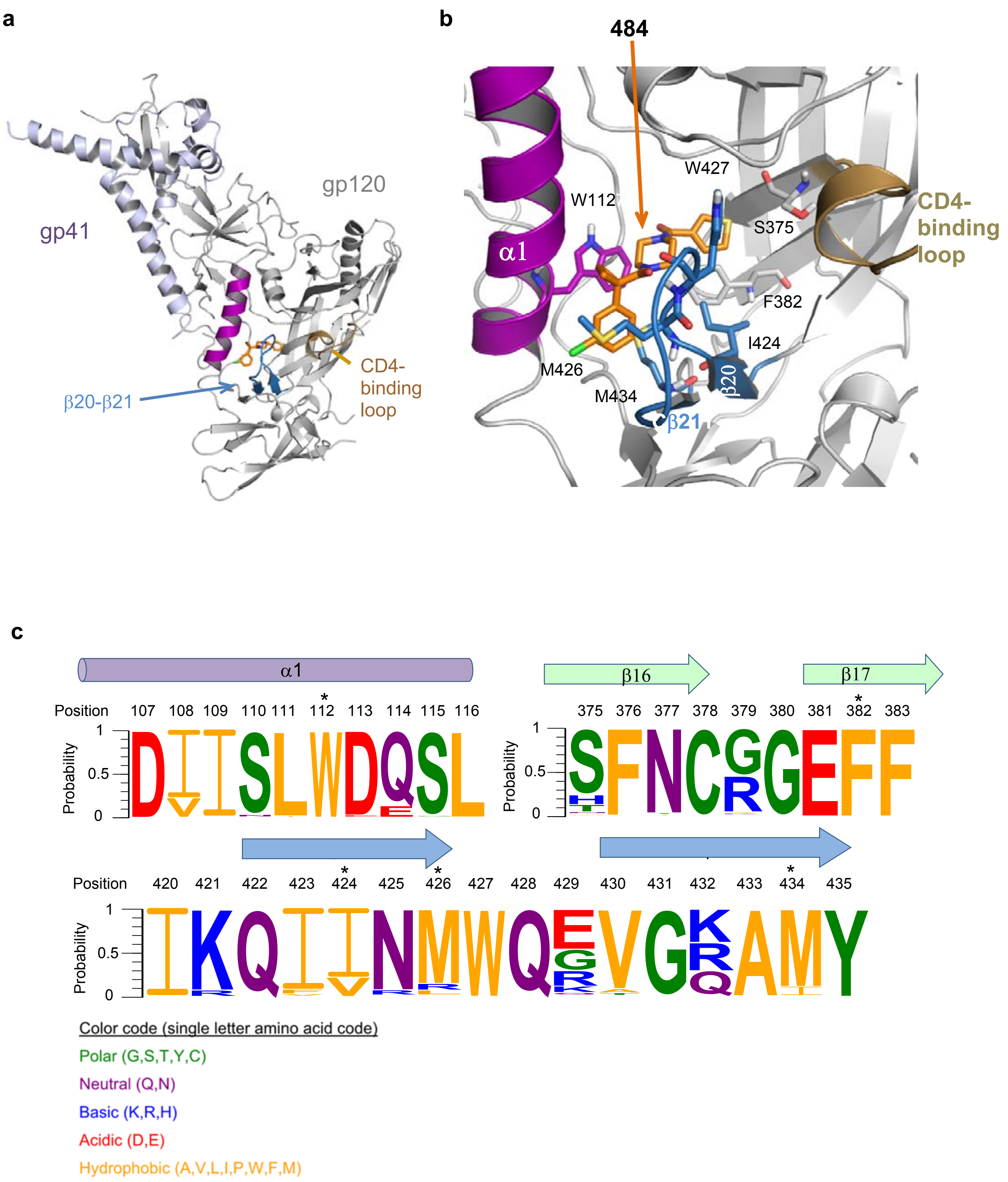
a
gp41
gp120
β20-β21
CD4-
binding
loop
b
484
W427
W112
S375
α1
CD4-
binding
loop
F382
I424
M426
M434
β20
β21
c
α1
β16
β17
Position
107 108 109 110 111 112 113 114 115 116
375 376 377 378 379 380 381 382 383
Probability
1
0.5
0
Position
420 421 422 423 424 425 426 427 428 429 430 431 432 433 434 435
Color code (single letter amino acid code)
Polar (G,S,T,Y,C)
Neutral (Q,N)
Basic (K,R,H)
Acidic (D,E)
Hydrophobic (A,V,L,I,P,W,F,M)

d

resistance ↑ **484** ↓ sensitive

HIV-1 isolate	α1	V1 Loop	V2 loop	β20-β21
	105 107	136 152	183 193	420 435
CAP201.2.0	HQD	TYNN----------GTNSTD	PLKNESES---QNFSEYIL	IRQIINMWQEVGRAMY
BG505	HTD	TNN----------ITDDMRG	QINENQGNRSNNSNKEYRL	IKQIINMWQRIGQAMY
KB9	HED	NITKNTTNLTSSSWGMMEEG	PVKN-------TSNTKYRL	IKQIINMWQKVGKAMY
ZM109	HED	AAH------------NESET	PLSSSDNS---SNSSLYRL	IKQIINMWQGVGRAMY
191859	HED	VNVINATGTEI----SSNST	PIDTENNN--NSSYNSYRL	IKQIINMWQGVGKAMY
Du422.1	HED	NISANANATA--TLNSSMNG	PLNGG-EH---NETGEYIL	IKQIINMWQEVGRAMY
191821	HED	KNKN-L-----------TKV	PINDNN-----STNTSYRL	IKQIINMWQGVGKAMY
ZM53M.PB12	QED	N--------------NATDG	PLDGR-N-----NSSEYRL	IKQIINMWQEVGRAMY
YU2	HED	RN---ATNTTSSSWETMEKG	PIDN----------ASYRL	IKQIINMWQEVGKAMY
190049	HED	KNNN------SKSNVTNEEI	QMD---------TNTSYRL	IKQIINMWQGVGKAMY
AD8	HED	RN---VTNIN-NSSE-GMRG	PIDN--------DNTSYRL	IKQIINMWQEVGKAMY
JR-FL	QED	NA---TNTTN-DSEGTMERG	PIDN--------NNTSYRL	IKQIINMWQEVGKAMY

Supplementary Figure 5. Modeling the binding of 484 to HIV-1 Env. (a) The binding site of **484** to one protomer of HIV-1 Env was estimated using molecular modeling and the cocrystal structure of BMS-626529 with the HIV-1$_{BG505}$ soluble gp140 SOSIP.664 trimer.[25] **(b)** Predicted **484**-interacting residues in the binding site of HIV-1 Env. **(c)** A LOGO plot describing the conservation of amino acids in HIV-1 gp120 regions comprising the proposed **484-**binding site. The amino acid sequence in specified regions was compared to the sequence present in HIV-1 isolates using QuickAlign (HIV sequence database, www.hiv.lanl.gov). Graphical representation of the distribution of amino acids at a specific position was generated with WebLogo.[26] The size of the letters represents the probability of finding a specific amino acid residue at that position. An asterisk indicates a residue proposed to contact **484**. **(d)** Alignment of four regions associated with HIV-1$_{JR-FL}$ resistance to 484 from the panel of isolates in Fig. 1A. Residues that differ from the sensitive HIV-1$_{JR-FL}$ are shown in red. A greater number of changes in the α1, V2 and β20-β21 regions is consistent with the profile of resistance. The DNA sequence for two isolates (191955-A4 and C30393_C3) was not available. The positions of the first and last residues in each region are shown above the sequences.

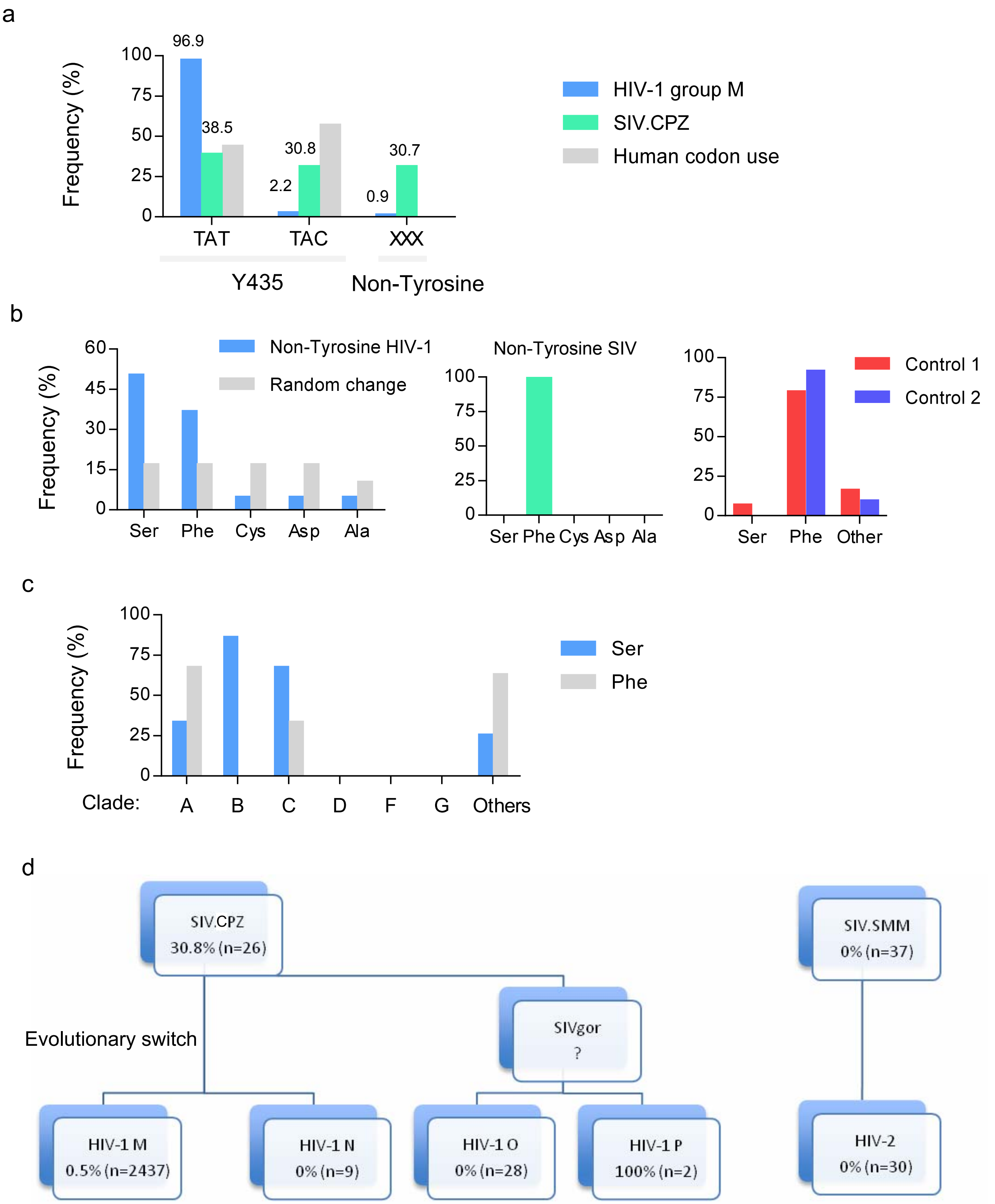
a
Frequency (%)
100
75
50
25
0
96.9
38.5
30.8
2.2
30.7
0.9
TAT
TAC
XXX
Y435
Non-Tyrosine
HIV-1 group M
SIV.CPZ
Human codon use
b
Frequency (%)
60
45
30
15
0
Non-Tyrosine HIV-1
Random change
Ser
Phe
Cys
Asp
Ala
Non-Tyrosine SIV
100
75
50
25
0
Ser Phe Cys Asp Ala
100
75
50
25
0
Control 1
Control 2
Ser
Phe
Other
c
Frequency (%)
100
75
50
25
0
Clade:
A
B
C
D
F
G
Others
Ser
Phe
d
SIV.CPZ
30.8% (n=26)
SIV.SMM
0% (n=37)
SIVgor
?
Evolutionary switch
HIV-1 M
0.5% (n=2437)
HIV-1 N
0% (n=9)
HIV-1 O
0% (n=28)
HIV-1 P
100% (n=2)
HIV-2
0% (n=30)

Supplementary Figure 6. Conservation and Evolution of Residue Tyr 435 in Primate Lentiviruses. (**a**) A significant bias in codon usage for the Tyr 435 amino acid in HIV-1 is not conserved in simian immunodeficiency virus (SIV). Tyr 435 is highly conserved (99.1%) in HIV-1 variants but exhibits only moderate conservation (69.3%) in SIV isolates. (**b**) Amino acid sequences of HIV-1 isolates that carry a residue other than tyrosine at position 435 are enriched in serine and then phenylalanine residues at this position. In contrast, substitution at Tyr 435 is almost exclusively to phenylalanine in sequences of SIV isolates (middle panel). Two randomly chosen control tyrosine residues in HIV-1 Env (residues 217 and 384) were also shown to exhibit preferential substitutions of phenylalanine (right panel). (**c**) For HIV-1 strains that do not carry Tyr 435, predominance of serine versus phenylalanine at position 435 is most notable in clade B and C. Clade A and all other HIV-1 clades show preferences for phenylalanine at this position. (**d**) Phylogenetic relationship between the different groups of primate lentiviruses. The percentage of the virus strains that carry amino acids other than tyrosine at position 435 are indicated. Evolution from SIV.CPZ to HIV-1 apparently imposed a stringent requirement for the tyrosine at position 435. Our mutagenesis results (Fig. 3e) raise the possibility that variation in residue 435 was driven by different requirements for Env responsiveness to CD4 binding.

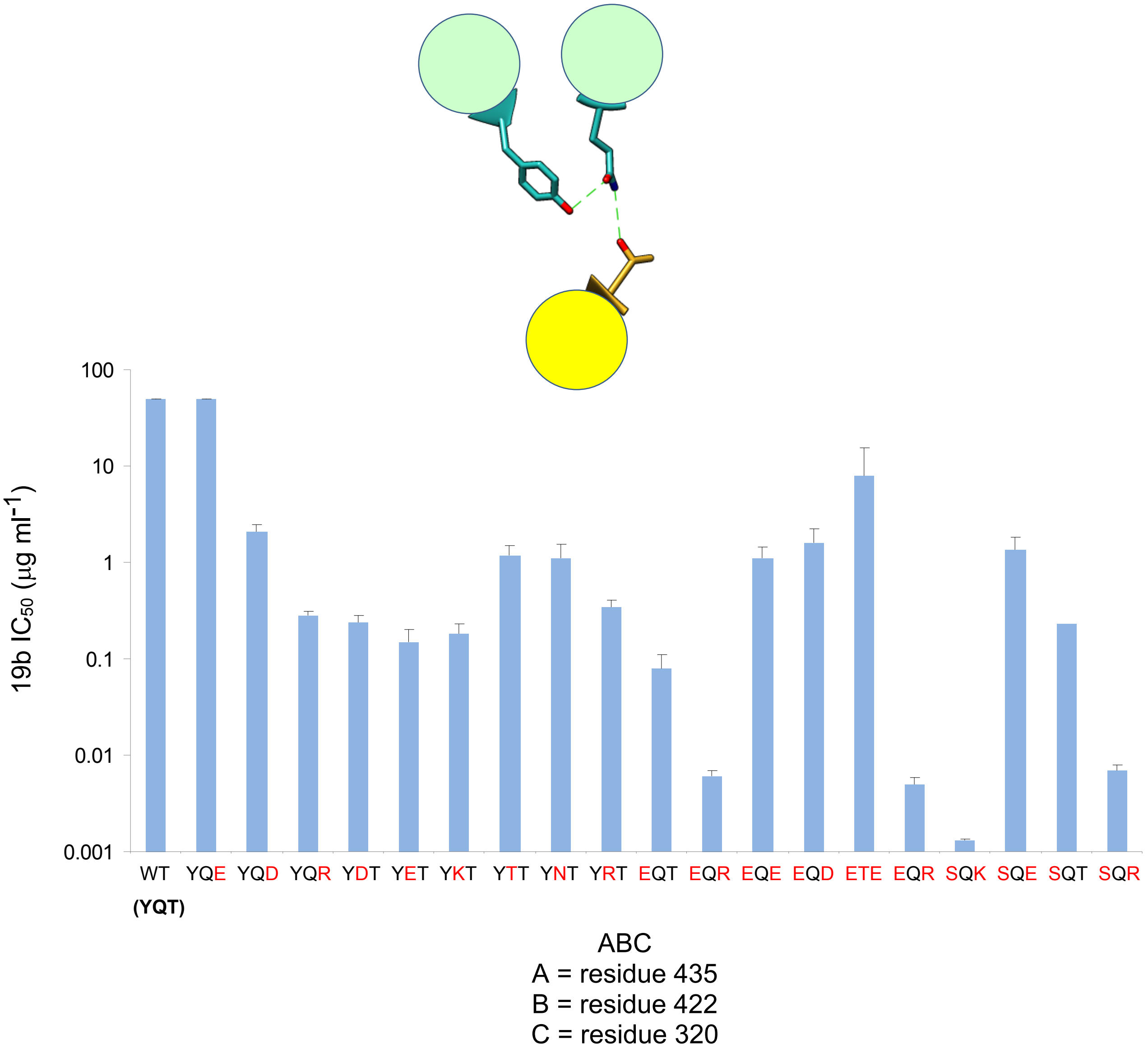

Supplementary Figure 7. Changes in gp120 residues 435, 422 and 320 strongly influence HIV-1$_{JR-FL}$ susceptibility to the 19b antibody. Residues 435 and 422 of the β20-β21 element are in close proximity to residue 320 of the V3 region in the HIV-1$_{BG505}$ soluble gp140 SOSIP.664 structure (PDB 4TVP). Potential contacts link the β20-β21 residues (Tyr 435 and Gln 422) to a residue in the base of the V3 loop (Thr 320). The inhibition by the 19b antibody of viruses with the indicated wild-type (WT) or mutant Envs is shown. Altered residues are shown in red letters. Inhibition data from 2-3 independent experiments, each performed in duplicate, were averaged. IC_{50} values were calculated by fitting the averaged data to the four-parameter logistic equation.

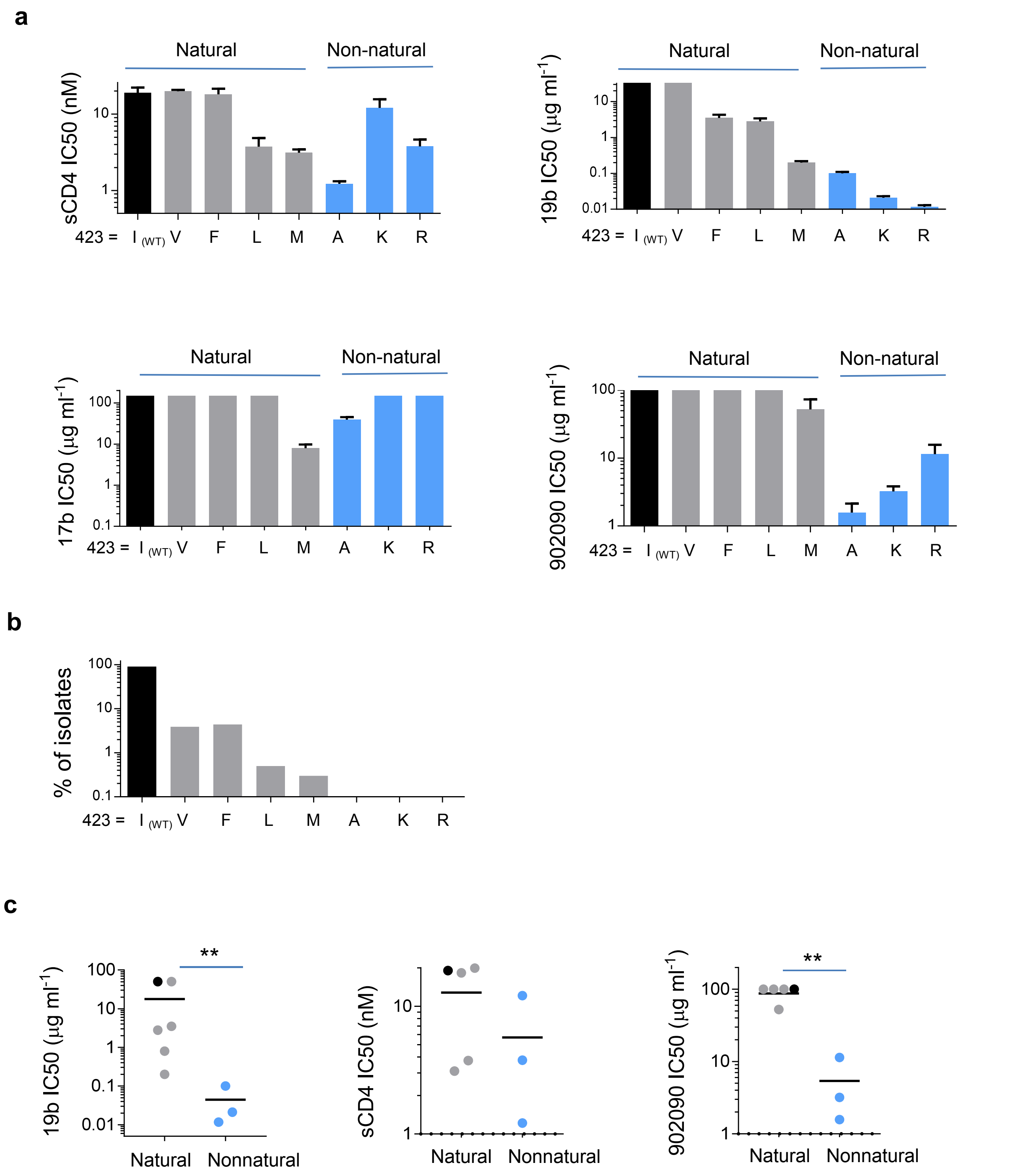

Supplementary Figure 8. The effect of different amino acid substitutions at residue 423 on the sensitivity of HIV-1$_{JR-FL}$ to Env ligands recognizing downstream conformations. (a) The indicated amino acid changes were introduced into HIV-1$_{JR-FL}$ Env and infection of the related pseudotyped virus was tested in the presence of the specified ligands. IC_{50} values were

calculated after fitting the inhibition curves to the four-parameter logistic equation. Substitutions that are found in primary HIV-1 isolates are labeled as natural and all others as non-natural. **(b)** Distribution of different amino acids among 2500 primary isolates at residue 423. **(c)** Comparison of the effect of natural and the non-natural substitutions at residue 423 on virus sensitivity to ligands that recognize downstream conformations. Color code is the same as that shown in panel (a). Mann-Whitney test was used to estimate statistical significance between the groups; ** , P value < 0.05. Sensitivity to 17b is shown in panel (a) but was not analyzed in panel (c) because the 17b epitope contains residue 423 (Rizzuto CD, Wyatt R, Hernandez-Ramos N, Sun Y, Kwong PD, Hendrickson WA, Sodroski J. 1998. A conserved HIV gp120 glycoprotein structure involved in chemokine receptor binding. Science 280:1949-1953). Therefore, lack of 17b activity against Ile 423 mutants may be related to poor antibody binding.

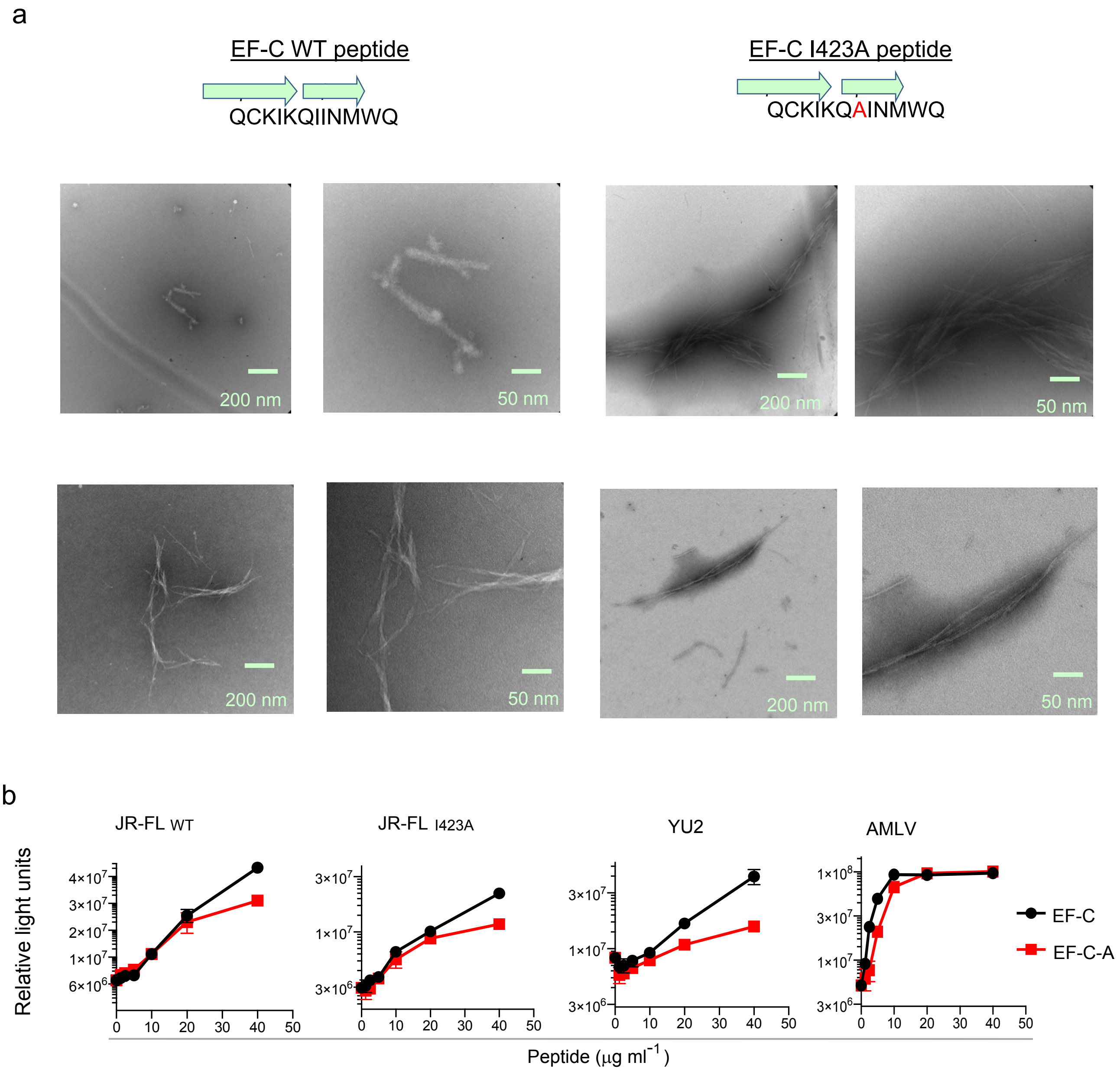

Supplementary Figure 9. Fibril formation by peptides derived from the β19 and β20 region of gp120 (Enhancing Factor C (EF-C) WT and EF-C I423A peptides). (**a**) Fibrils formed by the two 12-mer peptides were detected using electron microscopy. The peptides were diluted to 40 μg/ml, added to a formvar/carbon grid and negatively stained with NanoVan. Medium (x23,000 or x30,000) and high (x68,000) magnifications are shown. The peptide sequence and secondary structures within gp120 are shown (**b**) The enhancing effect of peptides from (a) on infectivity of different viruses.

Supplementary References

1. Scott, C.F., Jr. et al. Human monoclonal antibody that recognizes the V3 region of human immunodeficiency virus gp120 and neutralizes the human T-lymphotropic virus type IIIMN strain. *Proc Natl Acad Sci U S A* **87**, 8597-601 (1990).
2. Rizzuto, C.D. et al. A conserved HIV gp120 glycoprotein structure involved in chemokine receptor binding. *Science* **280**, 1949-53 (1998).
3. Wiehe, K. et al. Antibody light-chain-restricted recognition of the site of immune pressure in the RV144 HIV-1 vaccine trial is phylogenetically conserved. *Immunity* **41**, 909-18 (2014).
4. Pan, R., Gorny, M.K., Zolla-Pazner, S. & Kong, X.P. The V1V2 Region of HIV-1 gp120 Forms a Five-Stranded Beta Barrel. *J Virol* **89**, 8003-10 (2015).
5. Cavacini, L.A. et al. Human monoclonal antibodies to the V3 loop of HIV-1 gp120 mediate variable and distinct effects on binding and viral neutralization by a human monoclonal antibody to the CD4 binding site. *J Acquir Immune Defic Syndr* **6**, 353-8 (1993).
6. Posner, M.R., Cavacini, L.A., Emes, C.L., Power, J. & Byrn, R. Neutralization of HIV-1 by F105, a human monoclonal antibody to the CD4 binding site of gp120. *J Acquir Immune Defic Syndr* **6**, 7-14 (1993).
7. Shingai, M. et al. Antibody-mediated immunotherapy of macaques chronically infected with SHIV suppresses viraemia. *Nature* **503**, 277-80 (2013).
8. Wu, X. et al. Rational design of envelope identifies broadly neutralizing human monoclonal antibodies to HIV-1. *Science* **329**, 856-61 (2010).
9. Walker, L.M. et al. Broad and potent neutralizing antibodies from an African donor reveal a new HIV-1 vaccine target. *Science* **326**, 285-9 (2009).
10. Kong, R. et al. Fusion peptide of HIV-1 as a site of vulnerability to neutralizing antibody. *Science* **352**, 828-33 (2016).
11. Moldt, B. et al. Highly potent HIV-specific antibody neutralization in vitro translates into effective protection against mucosal SHIV challenge in vivo. *Proc Natl Acad Sci U S A* **109**, 18921-5 (2012).
12. Huang, J. et al. Broad and potent HIV-1 neutralization by a human antibody that binds the gp41-gp120 interface. *Nature* **515**, 138-42 (2014).

13. Huang, J. et al. Broad and potent neutralization of HIV-1 by a gp41-specific human antibody. *Nature* **491**, 406-12 (2012).
14. Stiegler, G. et al. A potent cross-clade neutralizing human monoclonal antibody against a novel epitope on gp41 of human immunodeficiency virus type 1. *AIDS Res Hum Retroviruses* **17**, 1757-65 (2001).
15. Platt, E.J., Wehrly, K., Kuhmann, S.E., Chesebro, B. & Kabat, D. Effects of CCR5 and CD4 cell surface concentrations on infections by macrophagetropic isolates of human immunodeficiency virus type 1. *J Virol* **72**, 2855-64 (1998).
16. Lin, P.F. et al. A small molecule HIV-1 inhibitor that targets the HIV-1 envelope and inhibits CD4 receptor binding. *Proc Natl Acad Sci U S A* **100**, 11013-8 (2003).
17. Madani, N. et al. Localized changes in the gp120 envelope glycoprotein confer resistance to human immunodeficiency virus entry inhibitors BMS-806 and #155. *J Virol* **78**, 3742-52 (2004).
18. Zhou, N. et al. Genotypic correlates of susceptibility to HIV-1 attachment inhibitor BMS-626529, the active agent of the prodrug BMS-663068. *J Antimicrob Chemother* **69**, 573-81 (2014).
19. Herschhorn, A. et al. A broad HIV-1 inhibitor blocks envelope glycoprotein transitions critical for entry. *Nat Chem Biol* **10**, 845-852 (2014).
20. Xiang, S.H. et al. Mutagenic stabilization and/or disruption of a CD4-bound state reveals distinct conformations of the human immunodeficiency virus type 1 gp120 envelope glycoprotein. *J Virol* **76**, 9888-99 (2002).
21. Kolchinsky, P., Kiprilov, E., Bartley, P., Rubinstein, R. & Sodroski, J. Loss of a single N-linked glycan allows CD4-independent human immunodeficiency virus type 1 infection by altering the position of the gp120 V1/V2 variable loops. *J Virol* **75**, 3435-43 (2001).
22. Tran, E.E. et al. Structural mechanism of trimeric HIV-1 envelope glycoprotein activation. *PLoS Pathog* **8**, e1002797 (2012).
23. Rasheed, M., Bettadapura, R. & Bajaj, C. Computational Refinement and Validation Protocol for Proteins with Large Variable Regions Applied to Model HIV Env Spike in CD4 and 17b Bound State. *Structure* **23**, 1138-49 (2015).

24. Wang, H. et al. Cryo-EM structure of a CD4-bound open HIV-1 envelope trimer reveals structural rearrangements of the gp120 V1V2 loop. *Proc Natl Acad Sci U S A* **113**, E7151-E7158 (2016).
25. Pancera, M. et al. Crystal structures of HIV-1 Env with small molecule-entry inhibitors BMS-378806 and BMS-626529. *Nat. Chem. Biol.*, in the press (2017).
26. Crooks, G.E., Hon, G., Chandonia, J.M. & Brenner, S.E. WebLogo: a sequence logo generator. *Genome Res* **14**, 1188-90 (2004).
